# Rechargeable Na-CO_2_ Batteries Starting from Cathode of Na_2_CO_3_ and Carbon Nanotubes

**DOI:** 10.1155/2018/6914626

**Published:** 2018-08-22

**Authors:** Jianchao Sun, Yong Lu, Hao Yang, Mo Han, Lianyi Shao, Jun Chen

**Affiliations:** Key Laboratory of Advanced Energy Materials Chemistry (Ministry of Education), College of Chemistry, Nankai University, Tianjin 300071, China

## Abstract

Na-CO_2_ batteries have attracted significant attentions due to their high energy density and effective utilization of greenhouse gas CO_2_. However, all reported Na-CO_2_ batteries employ excessive preloaded metal Na, which will lead to safety issues such as dendrite formation and short circuit. In addition, the charging mechanism of reported Na-CO_2_ batteries is not very clear. Here we report the Na-CO_2_ batteries, starting from the cathode of cheap Na_2_CO_3_ and multiwalled carbon nanotubes (CNTs). Due to the effective electron transfer and high reactivity, the decomposition of Na_2_CO_3_ and CNTs could take place under 3.8 V. The charging mechanism of 2Na_2_CO_3_ + C → 4Na + 3CO_2_ without any side reactions is revealed by in/ex situ techniques such as Raman, gas chromatograph, and optical microscope. Dendrite-free Na can quantitatively deposit on the Super P/Al anode because of large specific surface area and low nucleation barrier of the anode for Na plating. The batteries could deliver an energy density of 183 Wh kg^−1^ (based on the whole mass of the pouch-type batteries, 4 g) with stable cycling performance. This work reveals that safe rechargeable Na-CO_2_ batteries could be constructed by cheap Na_2_CO_3_ and multiwalled carbon nanotubes.

## 1. Introduction

Rechargeable Na-CO_2_ batteries have attracted a lot of interests because they can reuse the greenhouse gas CO_2_ to realize energy storage and conversion [1–4]. The reported rechargeable Na-CO_2_ batteries with liquid or quasi-solid state electrolyte employed metal Na and CO_2_ as the reactants. The discharge reaction can be described by 4Na + 3CO_2_ → 2Na_2_CO_3_ + C [3, 4]. However, Na-CO_2_ batteries still suffer from safety issues and self-discharge due to the use of excessive preloaded metal Na as anode. Metal Na would encounter dendrite formation or surface cracks during cycling process due to the uneven deposition of Na, leading to short circuit [5–7]. By contrast with metal Na, Na_2_CO_3_ and carbon are more stable, safer, and easier to get [8]. More importantly, the generated metal Na by the charging decomposition of Na_2_CO_3_ and carbon is stoichiometric. The batteries with stoichiometric metal Na will be safer than that with excessive preloaded metal Na. Therefore, designing dendrite-free rechargeable Na-CO_2_ batteries without preloaded metal Na is of great significance.

Due to its insulating properties, the decomposition of Na_2_CO_3_ will result in large polarization and inevitable side reactions such as electrolyte decomposition [9–11]. Thus, to develop rechargeable Na-CO_2_ batteries starting with Na_2_CO_3_ and carbon, the main issue is how to realize the decomposition of Na_2_CO_3_ and carbon under proper voltage range. To ensure fast kinetics such as effective electron transfer during the decomposition process, Na_2_CO_3_ should be evenly intertwined with highly conductive carbon and the particle size should be small [12–17]. Meanwhile, the reactivity of carbon materials also has a great influence on the decomposition process [18–21]. Furthermore, the decomposition mechanism of Na_2_CO_3_ and carbon in previous works is not very clear [3, 4]. It is also important to find out the source of CO_2_ in the charge process. In addition, to ensure Na metal is produced during charging and the generated stoichiometric metal Na does not form dendrite is also challenging [22–24].

**Figure 1:**
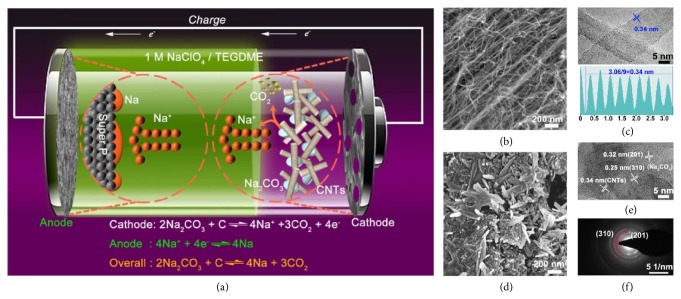
**The structure of rechargeable** **N** **a**-**CO**_2_** batteries. (a)** The scheme of Na-CO_2_ batteries starting from Na_2_CO_3_ and CNTs. SEM** (b)** and HRTEM** (c)** images of pristine CNTs with lattice distance of 0.34 nm. SEM** (d)** and HRTEM** (e)** images of prepared Na_2_CO_3_/CNTs composites.** (f)** The corresponding SAED pattern of** (e)**.

Here we report Na-CO_2_ batteries with Na-free architecture, which operate in pure CO_2_ atmosphere (1 atm) (Figure 1(a)). The cathode is composed of Na_2_CO_3_ and CNTs (multiwalled carbon nanotubes) with defects, where stick-shaped Na_2_CO_3_ (about 200-300 nm in length) are intertwined with CNTs closely and uniformly. The close and uniform structure is beneficial for promoting the decomposition of Na_2_CO_3_ and CNTs in proper voltage range (< 3.8 V). The anode is Super P/Al, which can effectively reduce the generation of Na dendrite and increase the Coulombic efficiency of the batteries because of the large specific surface area and low nucleation barrier for Na plating. The optimized electrolyte is 1 M NaClO_4_-tetraethylene glycol dimethyl ether (TEGDME) in view of its high voltage window (> 4.8 V versus Na^+^/Na) and high ionic conductivity (0.18 S m^−1^) ([Sec supplementary-material-1]). By means of various characterizations such as in situ Raman, gas chromatograph (GC), in situ optical microscopy, and nuclear magnetic resonance (NMR), we demonstrate that the charging reaction is 2Na_2_CO_3_ + C → 4Na + 3CO_2_ without any side reactions. After optimization, the dendrite-free Na-CO_2_ batteries show a relatively long cycling life (100 cycles) with cut-off capacity of 0.3 mAh cm^−2^. Remarkably, the pouch-type Na-CO_2_ batteries starting from Na_2_CO_3_ and CNTs deliver a total energy density of 183 Wh kg^−1^ (based on the whole mass of the pouch-type batteries, 4 g).

## 2. Results

### 2.1. Preparation of Na_2_CO_3_/CNTs Composite

To ensure the decomposition of cathode (Na_2_CO_3_ and carbon) under low voltage range, there are two key points which should be addressed properly. One is the combination status of Na_2_CO_3_ and carbon. The other is the carbon source, which acts as the conductive base and the reactant to participate in the charging reaction.

To guarantee uniform and close combination of Na_2_CO_3_ and carbon, we prepared the composites of Na_2_CO_3_ and carbon by a facile dissolution-crystallization method ([Sec supplementary-material-1]) [25]. The commercial CNTs have a porous cross-linking structure with the wall thickness of about 5 nm and spacing layers of 0.34 nm (Figures 1(b) and 1(c)). The outer walls of CNTs offer spaces for Na_2_CO_3_ to nucleate and anchor. Meanwhile, the inner walls act as conductive networks to promote electron transport easily [26, 27]. After the dissolution-crystallization process, we got the Na_2_CO_3_/CNTs composite, in which the stick-shaped Na_2_CO_3_ (about 200-300 nm in length) are intertwined with CNTs closely and uniformly (Figure 1(d)). Through the HRTEM images and the selected area electron diffraction (SAED), we can see the (201) and (310) planes of Na_2_CO_3_ and multiwall structure of CNTs in the composite (Figures 1(e) and 1(f)). In addition, we find that Na_2_CO_3_ crystal only grows on the outer walls of CNTs ([Sec supplementary-material-1]). The reason for this phenomenon is that the CNTs have less polarity and belong to superhydrophobic materials [28, 29]. There are some defects with hydrophilic groups (-OH, -COOH) in the outer wall of the CNTs, which is conducive to the nucleation and crystallization of water-soluble Na_2_CO_3_ on the outer walls of CNTs [30, 31]. However, the hydrophobicity of the inner walls prevents the water from entering.

We also used the same fabricating method to explore another four kinds of carbon materials, including reduced graphene oxide, Super P, single-walled carbon nanotubes, and double-walled carbon nanotubes. We finally chose multiwalled carbon nanotubes as the carbon source due to its high electrical conductivity, cross-linking structure, and suitable defects (optimization details can be seen in Supporting Information, Figures [Sec supplementary-material-1]-[Sec supplementary-material-1]).

### 2.2. Decomposition of Na_2_CO_3_/CNTs Cathode

After obtaining the Na_2_CO_3_/CNTs composite, we investigate the decomposition issue of Na_2_CO_3_/CNTs cathode by using Super P/Al as anode (the detailed optimization of anode can be seen in Figures [Sec supplementary-material-1]-[Sec supplementary-material-1]). In this part, we first optimized the content of Na_2_CO_3_ in the cathode and then revealed the charging mechanism thoroughly. The powder X-ray diffraction (XRD) patterns of pure CNTs, Na_2_CO_3_, and as-prepared Na_2_CO_3_/CNTs cathodes with different mass ratios are shown in [Sec supplementary-material-1]A. All diffraction peaks of pure Na_2_CO_3_ are well indexed with the standard XRD pattern of Na_2_CO_3_ (PDF no. 19-1130). In the XRD pattern of CNTs, a broad peak at 26.4° is assigned to the (002) crystal face. When the mass percentage of Na_2_CO_3_ in the cathodes is low (10, 20, and 30 wt%), there are no apparent diffraction peaks of Na_2_CO_3_, indicating that Na_2_CO_3_ are coated by CNTs. When the mass percentage of Na_2_CO_3_ further increases (40, 50, and 60 wt%), diffraction peaks of Na_2_CO_3_ emerge and gradually increase, which suggests that the grain size of Na_2_CO_3_ becomes large [32]. Due to the insulting properties of Na_2_CO_3_, charge transfer resistance of Na_2_CO_3_/CNTs cathodes becomes larger and larger when the mass percentage of Na_2_CO_3_ gradually increases ([Sec supplementary-material-1]B). When the mass percentage of Na_2_CO_3_ increases to 60 wt%, there is a jump in the impedance value, indicating that the content of Na_2_CO_3_ is too much to form uniform mixture.

**Figure 2:**
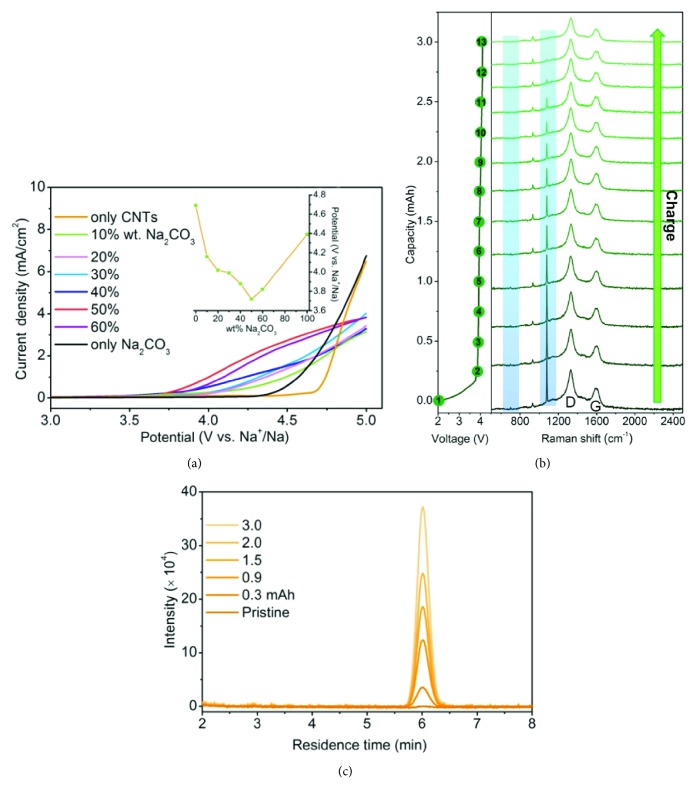
**Decomposition of** **N** **a**_2_ **C** **O**_3_/**CNTs cathode on charging process. (a)** LSVs of pure CNTs, 10%, 20%, 30%, 40%, 50%, and 60% weight ratio of Na_2_CO_3_ in Na_2_CO_3_/CNTs cathodes, and pure Na_2_CO_3_ with titanium powder (titanium powder acts as conductive additive) at the sweep rate of 1 mV s^−1^. The inset in** (a)** is the initial decomposition voltages of various cathodes.** (b)** The galvanostatic charge curve at 0.1 mA cm^−2^ with 13 selected points and corresponding in situ Raman spectra.** (c)** Evolution of CO_2_ characterized by GC.

Then, we explore the decomposition behavior of cathodes with different percentage of Na_2_CO_3_ by linear sweep voltammetry (LSV, Figure 2(a)). The results show that the decomposition voltages reduce first and then increase with the increased content of Na_2_CO_3_ in cathodes (the inset in Figure 2(a)). This result could be attributed to the following reasons. First, when there are only pure CNTs or Na_2_CO_3_, the charge reaction lacks another reactant, leading to a high charging voltage and resulting in the decomposition of the electrolyte. Note that we added 90 wt% titanium powders as conductive additive when we investigated the decomposition of pure Na_2_CO_3_ ([Sec supplementary-material-1]). Second, most of Na_2_CO_3_ are coated with CNTs when the content of Na_2_CO_3_ is low, so the electrolyte is difficult to spread to the surface of Na_2_CO_3_. When the amount of Na_2_CO_3_ increases to high value (60 wt%), the large particles of Na_2_CO_3_ cannot mix well with CNTs, resulting in poor conductivity. Therefore, a minimum decomposition voltage would be achieved when the content of Na_2_CO_3_ is intermediate. Here, a minimum decomposition voltage of 3.72 V was achieved when the content of Na_2_CO_3_ is 50 wt%, which should be originated from the best three-phase interface and the most uniform mixing degree with this content. In addition, the charge tests of cathodes were carried out, and the results are consistent with the LSV curves ([Sec supplementary-material-1]).

The interaction between Na_2_CO_3_ and CNTs in cathode is confirmed by X-ray photoelectron spectroscopy (XPS) ([Sec supplementary-material-1]). Compared with pure Na_2_CO_3_, the C1s spectra of CO_3_^2−^ in cathode shifted to the lower binding energy, indicating that the C atoms on the CNTs interact with the O atoms on the CO_3_^2−^ [33]. Meanwhile, the O atoms in the CO_3_^2−^ also reduce the strength of C-C on the CNTs, resulting in a new C-C peak at the 283.47 eV [34].

After optimizing the Na_2_CO_3_/CNTs cathode, we studied the charging mechanism through several methods. We first designed an in situ Raman coin cell with a 2 mm observation hole in the cathode cap to collect Raman signals from cathode in real time ([Sec supplementary-material-1]). Thirteen points were selected on charge profiles. As shown in Figure 2(b), the main peaks at 1080 and 700 cm^−1^ corresponding to Na_2_CO_3_ decrease little by little during charging process. In addition, the intensity of G and D band corresponding to CNTs also gradually decreases. The results indicate that the cathode can decompose in charging process.

Moreover, we employed gas chromatography (GC) measurements to monitor the gas generation during the charging process in pure argon atmosphere (1 atm) (Figure 2(c)). The results show that CO_2_ generates throughout the charging process. The retention time of CO_2_ in GC column is 6 minutes and no other new gases were detected. The practical evolution concentration of CO_2_ is 3.39 × 10^4^ ppm, which corresponds to 7.845 × 10^−5^ mol. This value is less than the theoretical one with 3.63 × 10^4^ ppm (8.393 × 10^−5^ mol, and detailed calculations can be found in the Supporting Information). The difference can probably be attributed to the reasons that CNTs has strong adsorption effect on CO_2_. In addition, CO_2_ also show certain solubility in TEGDME solvent [4, 35]. In order to prove that the CO_2_ is not produced by side reactions, we confirmed the components of electrolyte after charge by ^1^H and ^13^C NMR spectroscopy. The ^1^H and ^13^C NMR spectroscopy of the electrolyte have not changed after charge ([Sec supplementary-material-1]), indicating that the electrolyte did not decompose during the charging process.

We also characterized the morphological features of the cathode after charge by SEM ([Sec supplementary-material-1]). The results show that the surface of cathode becomes porous and the stick Na_2_CO_3_ disappears at the end of the charge process. The specific surface area of the cathode also increased from 27.498 m^2^ g^−1^ to 215.52 m^2^ g^−1^ after charge process ([Sec supplementary-material-1]). In the pristine cathode, Na_2_CO_3_ closely combined with carbon nanotubes (Figures [Sec supplementary-material-1]A and [Sec supplementary-material-1]B), resulting in a dense electrode. After charging, all the Na_2_CO_3_ are decomposed, and the original position of Na_2_CO_3_ is vacated. In addition, the carbon nanotube skeleton is well maintained ([Sec supplementary-material-1]). Therefore, the cathode becomes loose and porous, resulting in a significant increase in specific surface area. Furthermore, the charge transfer resistance (diameter of the semicircle) of the batteries also significantly reduced after full charge ([Sec supplementary-material-1]), indicating that the nonconductive Na_2_CO_3_ decomposed.

### 2.3. Na Generation

**Figure 3:**
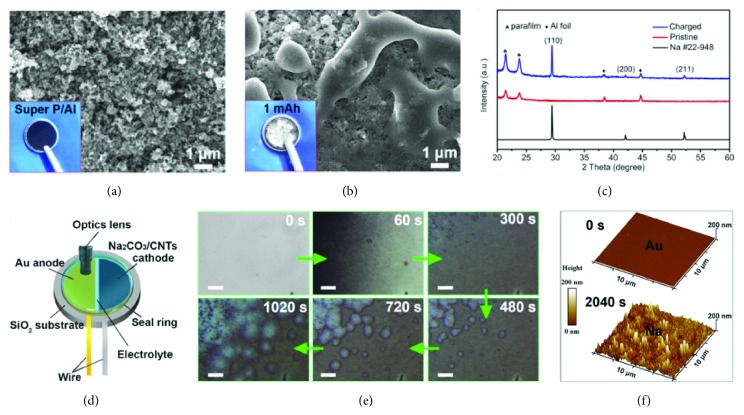
**Generation of Na on anode.** SEM images of** (a)** pristine Super P/Al anode and** (b)** the Super P/Al anode after charging to 1 mAh with Na coated on the surface. The insets in** (a)** and** (b)** are the corresponding photographs of the anode.** (c)** XRD patterns of Super P/Al anode before and after charge.** (d)** Schematic diagram of in situ optical microscope setup in liquid electrolyte.** (e)** In situ optical images for the process of Na formation.** (f)** AFM images of Na deposition on Au electrode at the time of 0 s and 2040 s. Scale bar: 40 *μ*m.

Combining the Super P/Al anode and optimized Na_2_CO_3_/CNTs cathode, we construct full batteries to characterize the generation of Na. SEM images of the pristine Super P/Al anode show that Super P with diameter of 30 nm are coated on the surface of Al (Figure 3(a) and [Sec supplementary-material-1]). Compared with the pristine anode, the electrode surface is covered by silver-white charging products after charging to 1 mAh (Figure 3(b)). XRD pattern of the charging products is shown in Figure 3(c). The peaks at 29.3°, 42.1°, and 52.2° are well matched with the (110), (200), and (211) crystal faces of Na (PDF no. 22-948), respectively. It can be seen from SEM images ([Sec supplementary-material-1]) that the metal Na has a smooth surface and disperses evenly, indicating that Super P plays a role in dispersing current density. The yield of Na is directly proportional to the charging capacity (1-3 mAh, [Sec supplementary-material-1]). When the charging capacity increased to 3 mAh, the surface of Super P was covered with a layer of Na.

In order to further observe the process of Na generation more visually, we designed an in situ optical microscopy setup to monitor the change of anode during charge process in real time (Figure 3(d)). The in situ optical microscopy setup includes an Au anode, a Na_2_CO_3_/CNTs cathode, a seal ring for sealing electrolyte, and an optics lens. We collected optical microscope images of the Au anode during the charge process at the sweep rate of 0.2 mV s^−1^ ([Sec supplementary-material-1]). Figure 3(e) shows a series of light-field optical microscope images, which successively present the morphology evolution of the Au electrode. The pristine Au electrode is clean and flat before charge voltage of 3.7 V (0 s). With the charging time going on, the surface of Au electrode gradually becomes dark (60 s). The XRD patterns ([Sec supplementary-material-1]) of Au electrode prove that the dark materials are metal Na. The entire surface of the Au electrode was covered by generated Na after 300 s. With further increase of charging depth, more Na deposited and covered the electrode layer by layer (480s, 720s, and 1020s). A video of Na deposition process can be seen in [Sec supplementary-material-1]. Moreover, we measured the surface height of Au electrode before and after charge through atomic force microscope (AFM) (Figure 3(f)). After charging for 2040 s, the height of Au electrode increases to 200 nm, indicating the thickness of deposited metal Na layer. These results demonstrate that the charging process accords with the following equation: 2Na_2_CO_3_ + C → 4Na + 3CO_2_.

### 2.4. Electrochemical Performance

After revealing the mechanism, we evaluate the electrochemical performance of the Na-CO_2_ batteries without preloaded metal Na in CO_2_ atmosphere (1 atm). The batteries firstly charged to produce Na. The voltage profile displays a flat plateau before charging to 4.6 mAh cm^−2^ ([Sec supplementary-material-1]), indicating that Na_2_CO_3_ and CNTs can decompose at low voltage. At the end of the charge process, the voltage curve starts to go up, demonstrating that all the Na_2_CO_3_ have almost broken down. The batteries show a maximal capacity of 5 mAh cm^−2^, which is very close to the theoretical value (calculation details can be seen in Supplementary Materials).

**Figure 4:**
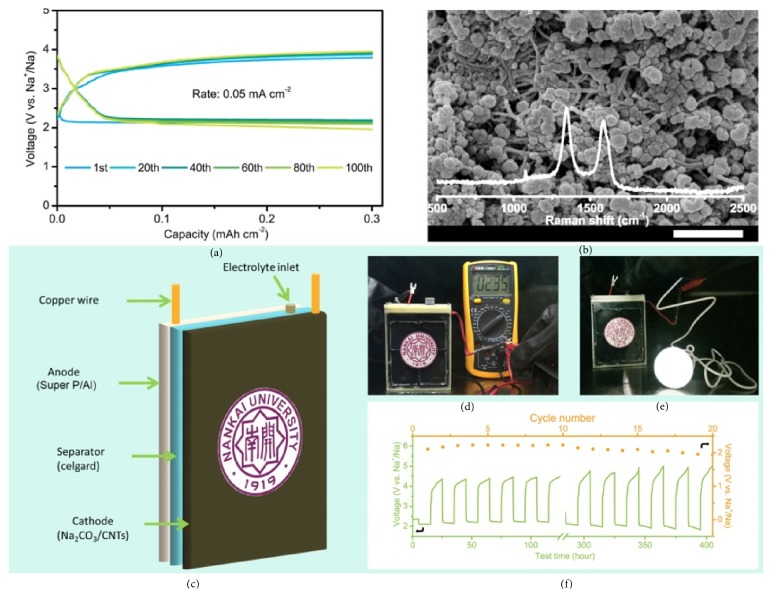
**Electrochemical performance of** **N** **a**-**CO**_2_** batteries without preloaded metal Na in pure CO**_2_** atmosphere with pressure of 1 atm. (a)** The cycling stability of Na-CO_2_ batteries with a cut-off capacity of 0.3 mAh cm^−2^ at current densities of 0.05 mA cm^−2^.** (b)** SEM images of the discharge products after first discharge process at the rate of 0.05 mA cm^−2^. The inset in** (b)** is the corresponding Raman spectrum.** (c)** Schematic illustration of the cell configuration composed of anode (Super P/Al), separator (Celgard), and cathode (Na_2_CO_3_/CNTs).** (d)** A digital photo of package battery with an open circuit voltage of 2.35 V.** (e)** A digital photo of package battery powering one-watt (1 W) light bulbs.** (f)** Cycling performance with a reversible capacity of 100 mAh at a current of 10 mA. Scale bar: 500 nm.

Subsequently, we evaluate cycling performance of the batteries at different current densities with the limited capacity at 0.3 mAh cm^−2^. At a current density of 0.05 mA cm^−2^, the charge potential of the batteries was maintained below 4 V after 100 cycles (Figure 4(a)). The results demonstrate an impressive stability of Na-CO_2_ batteries. As the current density increased to 0.10 and 0.15 mA cm^−2^, the batteries were capable of discharging/charging for over 50 cycles (Figures [Sec supplementary-material-1]A and [Sec supplementary-material-1]B). The excellent performance of the batteries shows that the Na generated by decomposition of Na_2_CO_3_ and CNTs can be used effectively. The surface morphology of Na coated Super P/Al anode after 50 cycles was studied by SEM ([Sec supplementary-material-1]). It can be seen that the Na covered surface of Super P/Al anode remains smooth and no Na dendrite is discovered, which suggests a uniform deposition/stripping process of Na on the surface of Super P/Al anode.

The morphological features of discharge products at different current densities were observed by SEM. We find that the current densities could affect the morphologies of the discharge products. As shown in Figure 4(b), round-shaped discharge products (~ 100 nm) form when the current density is 0.05 mA cm^−2^. As the current densities rise (0.10 and 0.15 mA cm^−2^), the discharge products become larger with rectangle shape (~ 200 nm) at 0.10 mA cm^−2^ and nanorod shape (~ 500 nm) at 0.15 mA cm^−2^ (Figures [Sec supplementary-material-1]A and [Sec supplementary-material-1]B). The changes in the morphology of the discharge products are similar to previous report [4]. Although the morphology of discharge products is different, we prove that Na_2_CO_3_ appear in all discharge products at different current densities through Raman characterization (the insets of Figure 4(b), Figures [Sec supplementary-material-1]A and [Sec supplementary-material-1]B). According to our previous work, the other discharge product is amorphous carbon that is mixed with sodium carbonate [3, 4].

We further fabricate pouch-type Na-CO_2_ batteries to assess their potential applications in energy storage. The pouch-type batteries are composed of Super P/Al (anode), Na_2_CO_3_/CNTs (cathode), and Celgard (separator) soaked in electrolyte (Figure 4(c) and [Sec supplementary-material-1]). During the charging process, the sodium ions are removed from Na_2_CO_3_ and in situ reduced to metal Na on the Super P/Al anode. When the charging process finished, the open circuit voltage of the batteries is 2.35 V (Figure 4(d)), which is consistent with the voltage of the common Na-CO_2_ batteries with preloaded metal Na. The pouch-type Na-CO_2_ batteries can light a LED bulb with 1 watt (Figure 4(e)). [Sec supplementary-material-1] is a video of the process that bulb is lit up. With cut-off voltage at 1.9 V, the initial discharge capacity of 350 mAh can be obtained at a current of 10 mA ([Sec supplementary-material-1]), corresponding to an energy density of 183 Wh kg^−1^ based on the whole mass of the pouch-type batteries (4 g). The energy density of the pouch-type Na-CO_2_ batteries is higher than that of commercial Li-ion batteries (150-180 Wh kg^−1^) [36]. The pouch-type Na-CO_2_ batteries exhibit stable cycling performance with an average discharge voltage of 2.13 V (Figure 4(f)). The results indicate the possibility of practical applications of Na-CO_2_ batteries which start from Na_2_CO_3_ and CNTs.

## 3. Discussion

In conclusion, we have successfully constructed dendrite-free rechargeable Na-CO_2_ batteries without preloaded metal Na. The batteries operate in pure CO_2_ atmosphere, consisting of Na_2_CO_3_/CNTs cathode, Super P/Al anode, and NaClO_4_/TEGDME electrolyte. The uniform Na_2_CO_3_/CNTs cathode with 50 wt% Na_2_CO_3_ could decompose under 3.8 V during the charge process. The Super P/Al anode is beneficial for dendrite-free Na plating/stripping (0.5 mAh cm^−2^, 100 cycles), showing higher and steadier Coulombic efficiency (98%) than that of pure Al anode. Combination of in situ Raman, GC, in situ optical microscopy, XRD, and NMR demonstrates that Na generate in the Super P/Al anode, and CO_2_ release in Na_2_CO_3_/CNTs cathode without any side reactions during initial charge process. The charging reaction could be described as 2Na_2_CO_3_ + C → 4Na + 3CO_2_. Due to effective use of the generated Na, the coin-type batteries are capable of charging/discharging for 100 cycles with a cut-off capacity of 0.3 mAh cm^−2^. Moreover, the pouch-type batteries deliver a large energy density of 183 Wh kg^−1^. Our work paves the way to construct safe and cheap Na-CO_2_ batteries without preloaded metal Na for practical energy storage applications.

## 4. Materials and Methods

### 4.1. Electrolyte Treatment

Tetraethylene glycol dimethyl ether (TEGDME) (Aladdin) is stilled by reduced pressure distillation method and stored with freshly activated molecular sieves (type 4 Å, beads 8-12 mesh, J&K). NaClO_4_ (anhydrous, Alfa Aesar) and NaCF_3_SO_3_ (anhydrous, Aladdin) are dried in a vacuum oven at 80°C for 24 hours before use. The electrolyte solutions of 1 M NaClO_4_/TEGDME and 1 M NaCF_3_SO_3_/TEGDME are prepared in a glove box filled with high-purity argon (O_2_ and H_2_O < 1 ppm).

### 4.2. Na_2_CO_3_/CNTs Cathode Preparation

In a typical preparation process, 50 mg commercial Na_2_CO_3_ and 40 mg CNTs dissolved in a mixed solvent including 4 mL ethanol and 4 mL water (1:1, v/v) under sonication for 2 hours. Then the solution was transferred to a culture dish and ultrasonic heated until solvent evaporation at 80°C. During the solvent volatilization process, Na_2_CO_3_ recrystallized and deposited on the surface of CNTs. After full evaporation of solvent, black Na_2_CO_3_/CNTs composite powder was obtained. The powder was characterized by XRD, XPS, SEM, and TEM. Then we rolled piece with Na_2_CO_3_/CNTs composite powder (90 wt%) and PTFE solution as binder (10 wt%), followed by drying in a vacuum oven at 80°C for 12 hours. Every piece weighs about 10~30 mg with a diameter of 14 mm. The other cathodes were prepared in the same way.

### 4.3. Super P/Al Anode Preparation

Conductive carbon black (TIMCAL Super P li) and polyvinylidene fluoride (PVDF) (9:1) were mixed with* N*-methylpyrrolidone (NMP). Slurry was then doctor bladed onto Al foil, followed by drying in a vacuum oven at 100°C for 10 hours to obtain Super P/Al anode with Super P mass loading of ~0.2 mg/cm^2^.

### 4.4. Batteries Assembly

In order to optimize the anode, the Na-Al or Na-Super P/Al batteries were assembled by stacking a metal sodium (12 mm in diameter), Celgard separator (16 mm in diameter) soaked with 60 *μ*L of electrolyte, and a Al foil or Super P/Al electrodes (14 mm in diameter) successively in a CR2032 coin-type battery. The full cells were also assembled in CR2032 coin cells with Super P/Al anode, Celgard separator soaked with 80 *μ*L of electrolyte, and the as-prepared Na_2_CO_3_/CNTs cathode. A hole is drilled in the cathode shell (4 mm in diameter) so that the CO_2_ can easily and quickly pass the cathode. Then, the battery is placed in a bottle filled with pure CO_2_ atmosphere. Pouch-type full cells are made up of one plastic case (8 × 9 cm^2^), a Super P/Al anode (7 × 6.8 cm^2^, 0.25 g), a piece of Celgard (8 × 7 cm^2^) with 2 mL electrolyte, and a Na_2_CO_3_/CNTs cathode (6 × 5.1 cm^2^, 1.6 g). The total mass of this pouch-type battery is 4 g. The pouch-type full cells run in a glove box with pure CO_2_ atmosphere.

## Data Availability

All data needed to evaluate the conclusions in the paper are present in the paper and/or the Supplementary Materials. Additional data related to this paper may be requested from the authors.

## Supplementary Materials

Supplementary Materials Figure S1. The electrochemical stability window and ionic conductivity of electrolyte. Figure S2. Schematic diagram of the fabrication process of Na_2_CO_3_/CNTs composites. Figure S3. TEM images of Na_2_CO_3_/CNTs cathode. Figure S4. The morphology characterization of various cathodes with different carbon sources and the comparision of charge voltage. Figure S5. Characterization of raw Na_2_CO_3_. Figure S6. The comparision of different carbon nanotubes. Figure S7. The optimized anode. Figure S8. Comparison of the sodium nucleation overpotential for Super P/Al and bare Al current collectors. Figure S9. SEM images of Na deposition (3 mAh) at different anodes. Figure S10. The optimization of Na_2_CO_3_/CNTs composites. Figure S11. EIS of mixed materials consisting of Na_2_CO_3_ and titanium powder (mass ratio of 1: 9). Figure S12. The charge profiles of pure CNTs, Na_2_CO_3_/CNTs, and pure Na_2_CO_3_ with titanium powder at current density of 0.1 mA cm^−2^. Figure S13. The X-ray photoelectron spectroscopy (XPS) of pure CNTs, pure Na_2_CO_3_ and Na_2_CO_3_/CNTs composites cathode of 50 wt% Na_2_CO_3_ content. Figure S14. The in-situ Raman battery. Figure S15. ^1^H NMR and ^13^C NMR spectra of electrolyte before and after charge. Figure S16. SEM images of Na_2_CO_3_/CNTs cathode after charge. Figure S17. The specific surface area (BET) of cathode. Figure S18. EIS of the battery before and after charge. Figure S19. SEM images of pristine Super P/Al anode. Figure S20. SEM images of Super P/Al electrode after charge. Figure S21. The photographs of Super P/Al anode with different charging capacity (0-3 mAh). Figure S22. A representative LSV curve of Na deposition in the in-situ tests with Na_2_CO_3_/CNTs as working electrode and Au as counter electrode. Figure S23. XRD patterns of the deposited Na on the Au electrode. Figure S24. The full charge profile of Na-CO_2_ batteries with 5 mAh cm^−2^ at 0.1 mA cm^−2^. Figure S25. The cycling stability of Na-CO_2_ batteries with a cut-off capacity of 0.3 mAh cm^−2^ at different current densities. Figure S26. SEM images of Na coated Super P/Al anode after 50 cycles, exhibiting a smooth surface. Figure S27. SEM images of the discharge products after first discharge process at different rates of (A) 0.10 mA cm^−2^ and (B) 0.15 mA cm^−2^. Figure S28. Photographs of Super P/Al anode and Na_2_CO_3_/CNTs cathode. Figure S29. Pouch-type battery performance. movie S1. Sodium deposition process. movie S2. Process that bulb is be lit up. References [31, 37–39].
