# Supplementary material for: Rechargeable Na-CO2 Batteries Starting from Cathode of Na2CO3 and Carbon Nanotubes
Source: Research (Wash D C). 2018 Aug 22;2018:6914626. doi: 10.1155/2018/6914626 (PMC6750045; doi:10.1155/2018/6914626)
Supplement: Supplementary Materials — Figure S1. The electrochemical stability window and ionic conductivity of electrolyte. Figure S2. Schematic diagram of the fabrication process of Na2CO3/CNTs composites. Figure S3. TEM images of Na2CO3/CNTs cathode. Figure S4. The morphology characterization of various cathodes with different carbon sources and the comparision of charge voltage. Figure S5. Characterization of raw Na2CO3. Figure S6. The comparision of different carbon nanotubes. Figure S7. The optimized anode. Figure S8. Comparison of the sodium nucleation overpotential for Super P/Al and bare Al current collectors. Figure S9. SEM images of Na deposition (3 mAh) at different anodes. Figure S10. The optimization of Na2CO3/CNTs composites. Figure S11. EIS of mixed materials consisting of Na2CO3 and titanium powder (mass ratio of 1: 9). Figure S12. The charge profiles of pure CNTs, Na2CO3/CNTs, and pure Na2CO3 with titanium powder at current density of 0.1 mA cm−2. Figure S13. The X-ray photoelectron spectroscopy (XPS) of pure CNTs, pure Na2CO3 and Na2CO3/CNTs composites cathode of 50 wt% Na2CO3 content. Figure S14. The in-situ Raman battery. Figure S15. 1H NMR and 13C NMR spectra of electrolyte before and after charge. Figure S16. SEM images of Na2CO3/CNTs cathode after charge. Figure S17. The specific surface area (BET) of cathode. Figure S18. EIS of the battery before and after charge. Figure S19. SEM images of pristine Super P/Al anode. Figure S20. SEM images of Super P/Al electrode after charge. Figure S21. The photographs of Super P/Al anode with different charging capacity (0-3 mAh). Figure S22. A representative LSV curve of Na deposition in the in-situ tests with Na2CO3/CNTs as working electrode and Au as counter electrode. Figure S23. XRD patterns of the deposited Na on the Au electrode. Figure S24. The full charge profile of Na-CO2 batteries with 5 mAh cm−2 at 0.1 mA cm−2. Figure S25. The cycling stability of Na-CO2 batteries with a cut-off capacity of 0.3 mAh cm−2 at different current densiti [file 6914626.f1.docx]

Supplementary Materials for

**Rechargeable Na-CO_2_ Batteries Starting from Cathode of Na_2_CO_3_ and Carbon Nanotubes**

Jianchao Sun, Yong Lu, Hao Yang, Mo Han, Lianyi Shao and Jun Chen*

Key Laboratory of Advanced Energy Materials Chemistry (Ministry of Education), College of Chemistry, Nankai University, Tianjin 300071, China.

*Corresponding author. E-mail: chenabc@nankai.edu.cn

**This PDF file includes:**

Materials characterization.

Electrochemical test.

CO_2_-evolution test.

Theoretical specific capacity calculation

Actual energy density calculation

Theoretical value of released CO_2_ in the charging process

fig. S1. The electrochemical stability window and ionic conductivity of electrolyte.

fig. S2. Schematic diagram of the fabrication process of Na_2_CO_3_/CNTs composites.

fig. S3. TEM images of Na_2_CO_3_/CNTs cathode.

fig. S4. The morphology characterization of various cathodes with different carbon sources and the comparision of charge voltage.

fig. S5. Characterization of raw Na_2_CO_3_.

fig. S6. The comparision of different carbon nanotubes.

fig. S7. The optimized anode.

fig. S8. Comparison of the sodium nucleation overpotential for Super P/Al and bare Al current collectors.

fig. S9. SEM images of Na deposition (3 mAh) at different anodes.

fig. S10. The optimization of Na_2_CO_3_/CNTs composites.

fig. S11. EIS of mixed materials consisting of Na_2_CO_3_ and titanium powder (mass ratio of 1: 9).

fig. S12. The charge profiles of pure CNTs, Na_2_CO_3_/CNTs, and pure Na_2_CO_3_ with titanium powder at current density of 0.1 mA cm^-2^.

fig. S13. The X-ray photoelectron spectroscopy (XPS) of pure CNTs, pure Na_2_CO_3_ and Na_2_CO_3_/CNTs composites cathode of 50 wt% Na_2_CO_3_ content.

fig. S14. The in-situ Raman battery.

fig. S15. ^1^H NMR and ^13^C NMR spectra of electrolyte before and after charge.

fig. S16. SEM images of Na_2_CO_3_/CNTs cathode after charge.

fig. S17. The specific surface area (BET) of cathode.

fig. S18. EIS of the battery before and after charge.

fig. S19. SEM images of pristine Super P/Al anode.

fig. S20. SEM images of Super P/Al electrode after charge.

fig. S21. The photographs of Super P/Al anode with different charging capacity (0-3 mAh).

fig. S22. A representative LSV curve of Na deposition in the in-situ tests with Na_2_CO_3_/CNTs as working electrode and Au as counter electrode.

fig. S23. XRD patterns of the deposited Na on the Au electrode.

fig. S24. The full charge profile of Na-CO_2_ batteries with 5 mAh cm^-2^ at 0.1 mA cm^-2^.

fig. S25. The cycling stability of Na-CO_2_ batteries with a cut-off capacity of 0.3 mAh cm^-2^ at different current densities.

fig. S26. SEM images of Na coated Super P/Al anode after 50 cycles, exhibiting a smooth surface.

fig. S27. SEM images of the discharge products after first discharge process at different rates of (A) 0.10 mA cm^-2^ and (B) 0.15 mA cm^-2^.

fig. S28. Photographs of Super P/Al anode and Na_2_CO_3_/CNTs cathode.

fig. S29. Pouch-type battery performance.

movie S1. Sodium deposition process.

movie S2. Process that bulb is be lit up.

References (*37-40*)

**Materials characterization.** Chemical composition is confirmed by Powder X-ray diffraction (XRD) patterns with a Rigaku MiniFlex600 X-ray diffractometer with Cu Kα radiation (λ=1.54 Å) and X-ray photoelectron spectroscopy (XPS, Perkin Elmer PHI 1600 ESCA system). The electrodes are sealed by using parafilms for the XRD test. The Raman and in-situ Raman spectra are collected at room temperature by using a Thermo-Fisher Scientific (excitation wavelength, 532 nm). Fourier transform infrared (FT-IR) spectroscopy is recorded with a FT-IR-650 spectrometer at a resolution of 2 cm^-1^. Scanning electron microscopic (SEM) images are collected using a JEOL JSM-7500F in field emission scanning electron microscope (operating voltage, 5 kV). TEM and high-resolution TEM images are taken with a Philips Tecnai G2F-20 (acceleration voltage, 200 kV). H spectrum (^1^H-NMR) and C spectrum (^13^C NMR) were tested by Bruker AVANCE 400 to characterize the electrolyte composition, confirming the stability of electrolyte during charge process. In situ optical microscopy and AFM experiments were conducted with a commercial AFM (Bruker Multimode 8) at room temperature.

**Electrochemical test.** The LAND CT2001A battery test instrument is used to the galvanostatic discharge/charge tests. The specific capacity and current density are based on the area of cathode (1.5386 cm^2^). Linear sweep voltammograms (LSVs) of cathodes at the sweep rate of 1 mV s^-1^ with the potential window of 3.0−5.0 V (vs Na^+^/Na). Cyclic voltammograms (CVs) of electrolyte are measured on a Parstat 263A electrochemical workstation (AMTECT Company, USA) in the potential window of -0.25−5.0 V (vs Na^+^/Na). Electrochemical impedance spectroscopy (EIS) is conducted on Parstat 2273A potentiostat/galvanostat workstation (AMETEK Company) in the frequency range from 100 kHz to 10 mHz.

**CO_2_-evolution test.** Gas evolution is tested by GC-2010 Plus (SHIMADZU) with ﬂame ionization detectors, stabilwax capillary column (30 m × 0.32 mm, ﬁlm thickness of 0.5 μm) helium as carrier gas, and ion source temperature of 250 °C. The charging atmosphere was identiﬁed based on retention indices, which are determined by the compounds retention time in capillary column. The battery assembly in this study is based on a CR2032 coin type battery with a hole in the cathode shell. The volume of bottle which contained the battery is 50 mL. In the original state, the bottle was filled with argon. The practical CO_2_ concentration was calibrated by a sample gas which containing 5 ppm CO_2_. When charged to 3 mAh, the actual evolution concentration of CO_2_ is 3.39 × 10^4^ ppm, which corresponds to 7.845 × 10^-5^ mol.

**Theoretical specific capacity calculation**

The charging reaction of Na-CO_2_ battery can be described as equation (1):

$C+2N\text{a}_{2}CO_{3}\to3CO_{2}+4Na$ (1)

When charged to 1 mAh, the electron transfer mole number (n) can be calculated in the following method:

Q=1 mAh = 1 mA × 3600 s =3.6 C

The electron transfer mole number (n) was obtained by equation:

$\text{n}=\frac{Q}{F}$ (2)

where Q represents quantity of electricity; F is Faraday constant (96485 C⋅mol^-1^). So, n= 3.73 × 10^-5^ mol. The mole number of transferred electron is exactly equal to the mole number of produced sodium. Therefore, the mole number of decomposed Na_2_CO_3_ and carbon can be calculated using the following the equation:

$\frac{2}{\text{x}}=\frac{4}{3.73\times10^{-5}mol}$ (3)

$\frac{1}{\text{y}}=\frac{4}{3.73\times10^{-5}mol}$ (4)

where 2, 1 and 4 represent the coefficient of Na_2_CO_3_, carbon and sodium in the equation of charging reactions, respectively. The x and y are the mole number of decomposed Na_2_CO_3_ and carbon, respectively. So, x= 1.865 × 10^-5^ mol, y= 9.33 × 10^-6^ mol. The corresponding mass of decomposed Na_2_CO_3_ and carbon are 1.98 mg and 0.11 mg, respectively. Therefore, charging to 1mAh corresponds to decomposing 1.98 mg Na_2_CO_3_. The Na_2_CO_3_/CNTs cathode weighs about 10~30 mg (due to the different thickness of the cathode) with a diameter of 14 mm. When Na_2_CO_3_ is 50% of the total mass of cathode, the theoretical specific capacity (based on the area of cathode) is about 1.6-5.0 mAh cm^-2^.

**Actual energy density calculation**

The energy density (E_d_) of pouch-type battery can be calculated by equation (5):

$E_{\text{d}}=(E\times C)/M$ (5)

Where E (2.096 V) is the discharge voltage; C (350 mAh) is the capacity of full discharge; M (4 g) is the mass of whole pouch-type battery. The obtained actual energy density is 183 Wh kg^-1^.

**Theoretical value of released CO_2_ in the charging process**

When charged to 1 mAh, the mole number of produced sodium is 3.73 × 10^-5^ mol (the detailed calculation can be seen in the above part of “Theoretical specific capacity calculation”). So, the amount of Na is 1.119 × 10^-4^ mol when charging to 3 mAh. Based on the equation (1), the amount of CO_2_ produced in charging reaction can be calculated by following equation:

$\frac{3}{\text{z}}=\frac{4}{1.119\times10^{-4}mol}$ (6)

where 3 and 4 represent the coefficient of CO_2_ and sodium in the equation (1), respectively. So, the amount of produced CO_2_ (z in the equation 6) in charging reaction is 8.3925 × 10^-5^ mol. The volume of bottle which contained the battery is 50 mL. According to the ideal gas Law, the account of Ar in this bottle is 2.23× 10^-3^ mol. Therefore, the theoretical value of CO_2_ (ppm) in this bottle can be calculated by the following equation:

$A=\frac{n_{co_{2}}}{n_{co_{2}}+n_{Ar}}$ (7)

Where A represents the theoretical value of released CO_2_ (ppm), the theoretical value of CO_2_ is 36300 ppm.


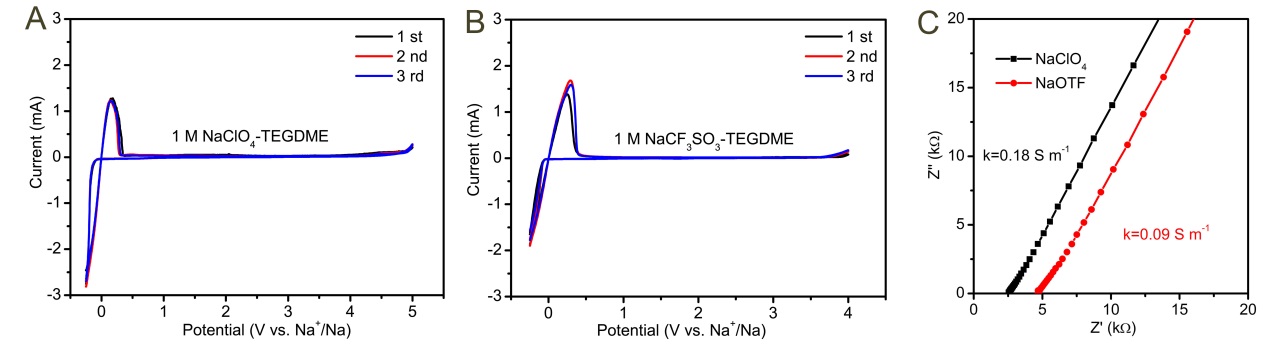


**fig. S1. The electrochemical stability window and ionic conductivity of electrolyte.** **(A)** 1 M NaClO_4_-TEGDME (G4) electrolyte. **(B)** 1 M NaCF_3_SO_3_-G4 electrolyte. **(C)** The ionic conductivity of 1 M NaClO_4_-G4 and 1 M NaCF_3_SO_3_-G4 electrolyte.

The electrochemical stability of electrolyte is an important factor in Na-CO_2_ batteries. Tetraethylene glycol dimethyl ether (TEGDME) was chosen as solvent by reasons of its low volatile (boiling point, 275 °C) (*37, 38*). Two kinds of electrolytes separately containing sodium salt of NaClO_4_, NaCF_3_SO_3_ were investigated. The electrochemical stability window of 1 M NaClO_4_-G4 electrolyte was tested by cyclic voltammetry with titanium foil as working electrodes at the scan rate of 1 mV s^-1^ between -0.25 and 5.0 V. The electrochemical stability window of 1 M Na NaCF_3_SO_3_-G4 electrolyte was tested by cyclic voltammetry with titanium foil as working electrodes at the scan rate of 1 mV s^-1^ between -0.25 and 4.0 V. The results show that the decomposition of 1M NaClO_4_-TEGDME electrolyte begins at around 4.85 V (vs. Na^+^/Na) with low current. A wide voltage window is beneficial for fully decomposition of Na_2_CO_3_ and CNTs in charging process. By contrast, the decomposition of 1 M NaCF_3_SO_3_-TEGDME starts from 3.8 V. Moreover, 1 M NaClO_4_-TEGDME owns higher ionic conductivity (k = 0.18 S m^‒1^) than 1 M CF_3_SO_3_Na-TEGDME (k = 0.09 S m^-1^). Based on higher ionic conductivity and wider electrochemical stability window, 1M NaClO_4_-TEGDME was used as the electrolyte.


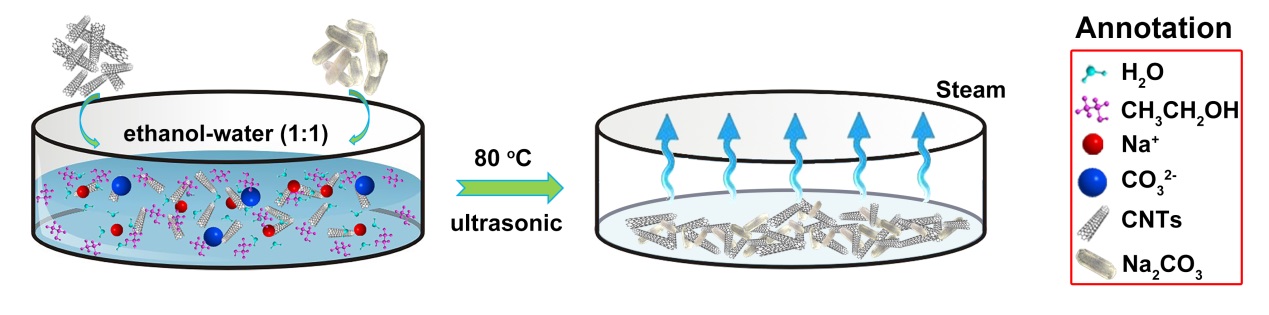
**fig. S2. Schematic diagram of the fabrication process of Na_2_CO_3_/CNTs composites.**

As shown in fig. S2 (taking CNTs as an example), we first dissolved Na_2_CO_3_ in the well-dispersed CNTs solution of ethanol-water (v:v, 1:1). Then the solution was ultrasonic at 80 °C until the solvent was evaporated. The function of water in solvent is dissolving Na_2_CO_3_ and dispersing CNTs. Because of ethanol-insolubility of sodium carbonate and high volatility of ethanol, ethanol can accelerates the volatilization of solvents and nucleation of Na_2_CO_3_. After evaporation of the solvents, CNTs could form three-dimensional conductive networks by self-assembly, and simultaneously Na_2_CO_3_ could recrystallize around the surface of CNTs.


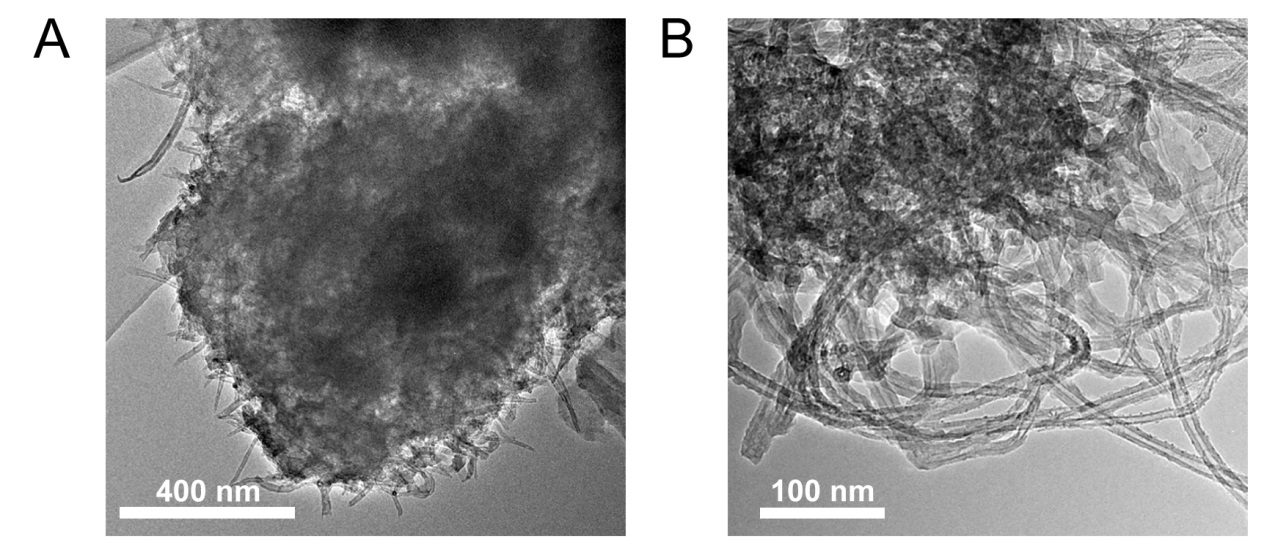


**fig. S3. TEM images of Na_2_CO_3_/CNTs cathode.**


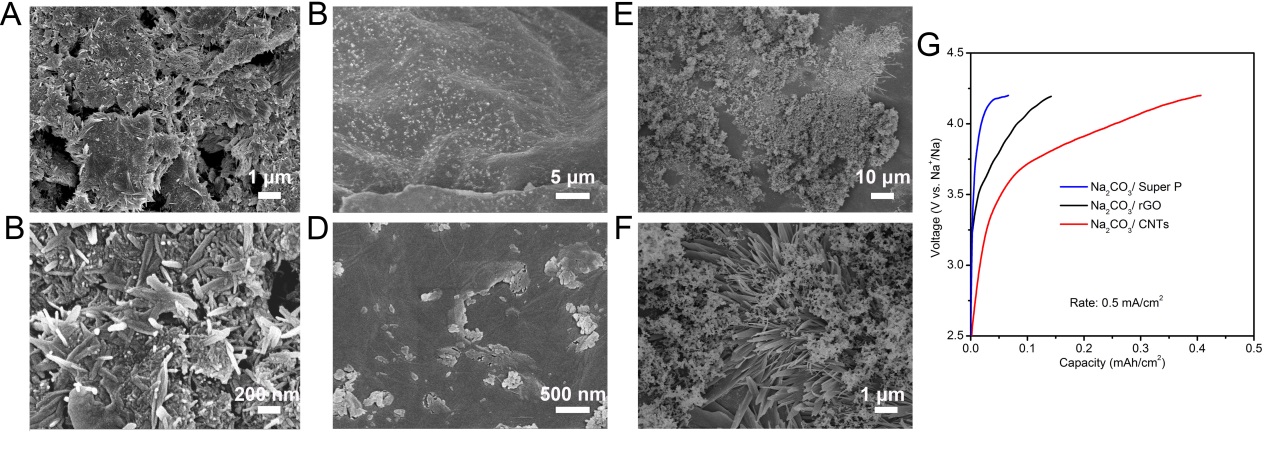


**fig. S4. The morphology characterization of various cathodes with different carbon sources and the comparision of charge voltage.** SEM images of **(A)** and **(B)** Na_2_CO_3_/CNTs, **(C)** and **(D)** Na_2_CO_3_/rGO, **(E)** and **(F)** Na_2_CO_3_/Super P. **(G)** Charge curves with different kinds of cathodes at current density of 0.5 mA cm^-2^.

Through the same procedure (carbon: Na_2_CO_3_, 1:1, w/w), the cathodes with different kinds of carbon are prepared. It can be seen that the cathode prepared by multi-walled carbon nanotubes (CNTs) is the most homogeneous, in which stick-shaped Na_2_CO_3_ (about 200~300 nm in length) are intertwined with CNTs closely and uniformly. In addition, the size of Na_2_CO_3_ particles is the smallest in Na_2_CO_3_/CNTs cathode. This may be due to the three-dimensional conductive network structure assembled by CNTs, which is more beneficial for the uniform nucleation of Na_2_CO_3_. Moreover, the size of Na_2_CO_3_ crystals prepared by this method is much smaller than that of raw Na_2_CO_3_ (fig. S4). The charge curves with different cathodes were tested by LAND battery test instrument with Na as counter electrode at the current density of 0.5 mA cm^-2^ with cut-off voltage of 4.2 V. The results show that the charging voltage of Na_2_CO_3_/Super P electrode quickly reaches 4.2V. The performance of Na_2_CO_3_/CNTs is far better than that of Na_2_CO_3_/rGO electrode.


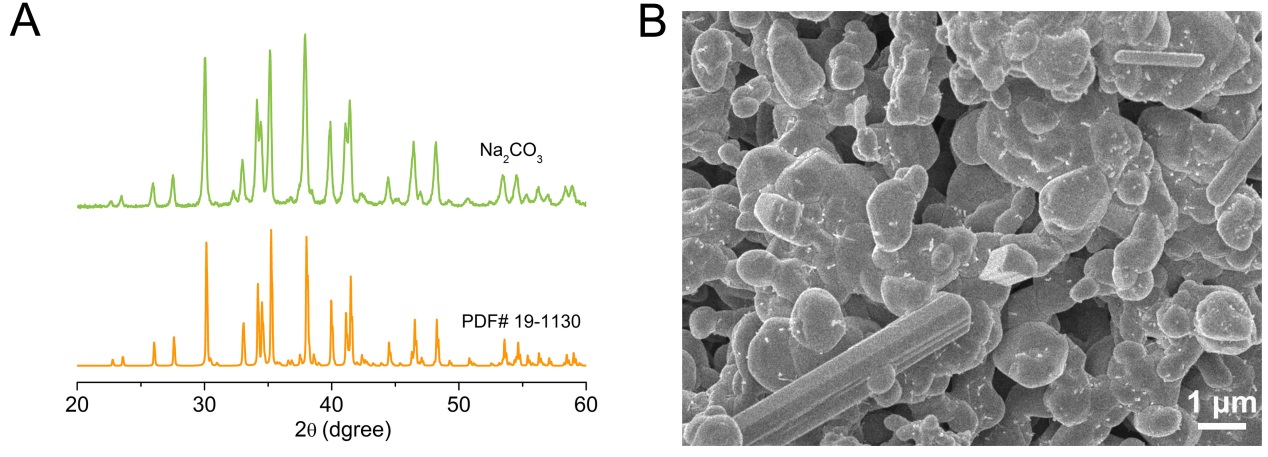


**fig. S5. Characterization of raw Na_2_CO_3_.** **(A)** XRD pattern, and **(B)** SEM image of commercial Na_2_CO_3_.


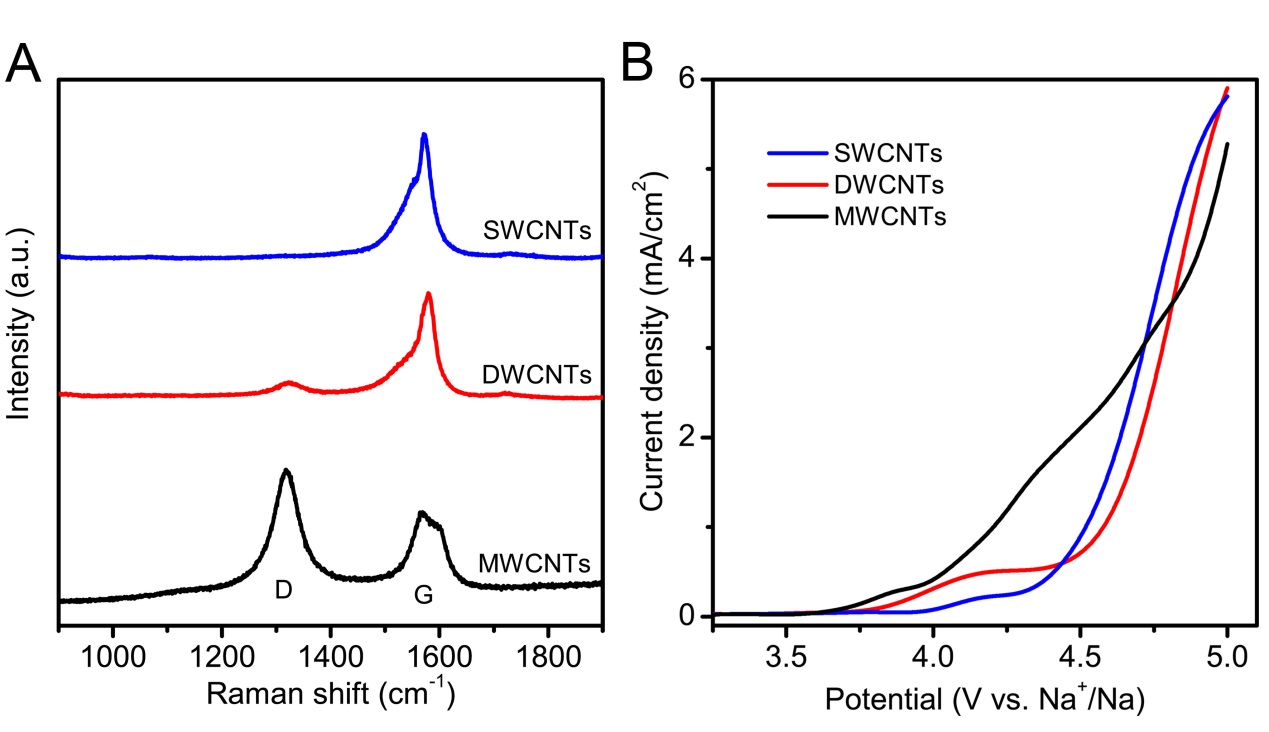


**fig. S6. The comparision of different carbon nanotubes.** **(A)** Raman spectra of SWCNTs, DWCNTs and MWCNTs. **(B)** Linear sweep voltammetry (LSV) curves of Na_2_CO_3_/SWCNTs, Na_2_CO_3_/DWCNTs and Na_2_CO_3_/MWCNTs cathodes at the sweep rate of 1 mV s^-1^.

Supplementary fig. S5A shows the Raman spectra of single-walled carbon nanotubes (SWCNTs), double-walled carbon nanotubes (DWCNTs), and multi-walled carbon nanotubes (MWCNTs). The results show that there is no D band of SWCNTs, indicating that the carbon is connected with the ideal hexagon without disordered carbon (*19*). The Raman spectrum of DWCNTs is similar to that of SWCNTs, but small amount of disoreder carbon appear (D band, defect-induced mode) (*31*). By constrast, the spectrum of the MWCNTs is different from those of SWCNTs and DWCNTs. The I_D_/I_G_ value of MWCNTs is as high as 1.34, which is the characteristic of disordered carbonaceous structures (*20*).

In order to explore which carbon nanotubes is best for charging, anodic LSV was employed with the above materials as the cathode (fig. S5B). The results show that the decomposition voltage of Na_2_CO_3_/MWCNTs cathode is the lowest. This result may be due to the following reasons. Firstly, disordered carbon existed in the MWCNTs and DWCNTs, and the decomposition of disordered carbon may require lower energy (*39*). In addition, due to the presence of many layers, the outer walls of MWCNTs can participate in the reaction, while the inner walls act as conductive network to allow electrons transport easily. Meanwhile, considering the price of materials, we chose MWCNTs rather SWCNTs and DWCNTs as carbon sources. Note that CNTs represents MWCNTs for brevity in the main text.


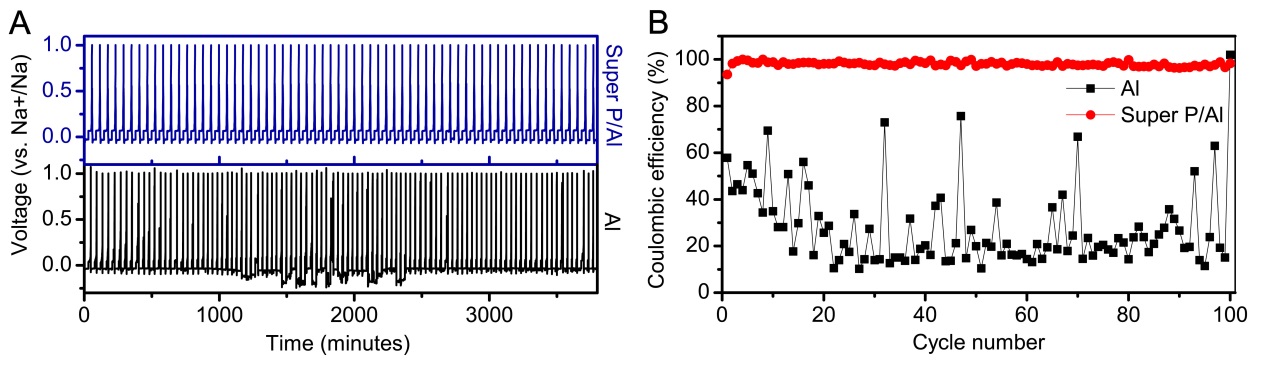


**fig. S7. The optimized anode.** **(A)** Cycling performance of bare Al and Super P/Al current collectors at 1 mA cm^-2^ (plating time: 0.5 hour for each cycle), and **(B)** corresponding Coulombic efficiency for over 100 plating/stripping cycles.

Prior to characterizing the generation of Na in the anode during the charge process, we compared two kinds of anode materials. They are pure Al and Super P/Al (Al with Super P coating layers) (*23, 40*). We selected Al substrate instead of frequently-used Cu foil because Al is more stable at higher potential, which is helpful for stability of the batteries system. We studied the galvanostatic cycling performance of Super P/Al and bare Al foil in half cells, using metal Na as reference electrode and 1 M NaClO_4_/TEGDME as electrolyte at a current density of 1 mA cm^-2^ with a capacity limitation of 0.5 mAh cm^-2^ (fig. S6A). The results show that Super P/Al anode exhibits more stable plating/stripping performance and higher Coulombic efficiency (98%) than those of pure Al electrode (fig. S6B). These elevated performance can be attributed to the increased specific surface area provided by Super P (an increase of about 200 times in surface area for a 200 μg cm^-2^ carbon layer), which can disperse current density of the electrode.


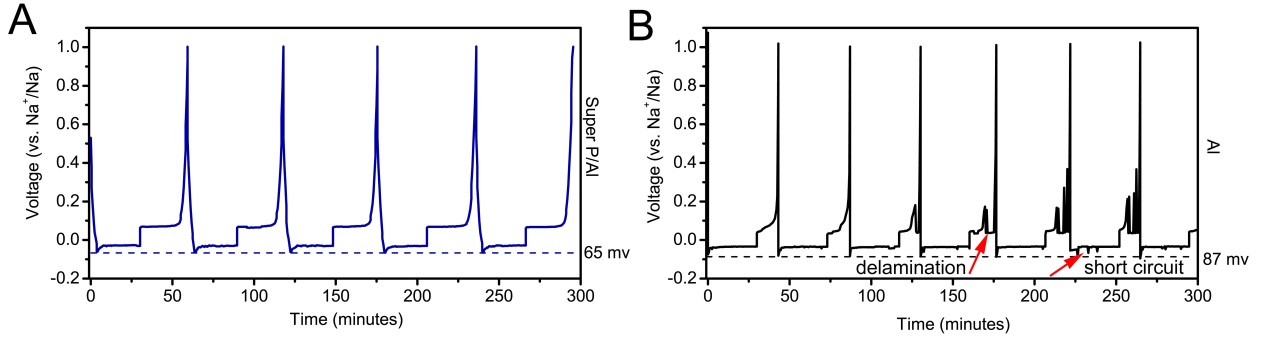


**fig. S8. Comparison of the sodium nucleation overpotential for Super P/Al and bare Al current collectors.**

Comparing the plating process for Super P/Al and bare Al current collectors, we find that nucleation potential reduces from 87 to 65 mV by the Super P layer. A reduction in the nucleation barrier is beneficial for facilitating smooth deposition and high-rate performance. In the initial cycles, we see a failure in the Al electrodes, where a stripping process is cut short owing to delamination of the metal Na from the current collector. Such phenomenon did not take place in the Super P/Al electrodes due to the improved mechanical stability achieved by utilizing a carbon nucleation layer, which provided three-dimensional interface. During the plating process, we also see that the bare Al electrode exhibits signs of short circuit. The phenomenon can be attributed to the uneven plating due to the high nucleation barrier. By contrast, the Super P/Al electrodes show more stable plating and stripping with higher Coulombic efficiency.


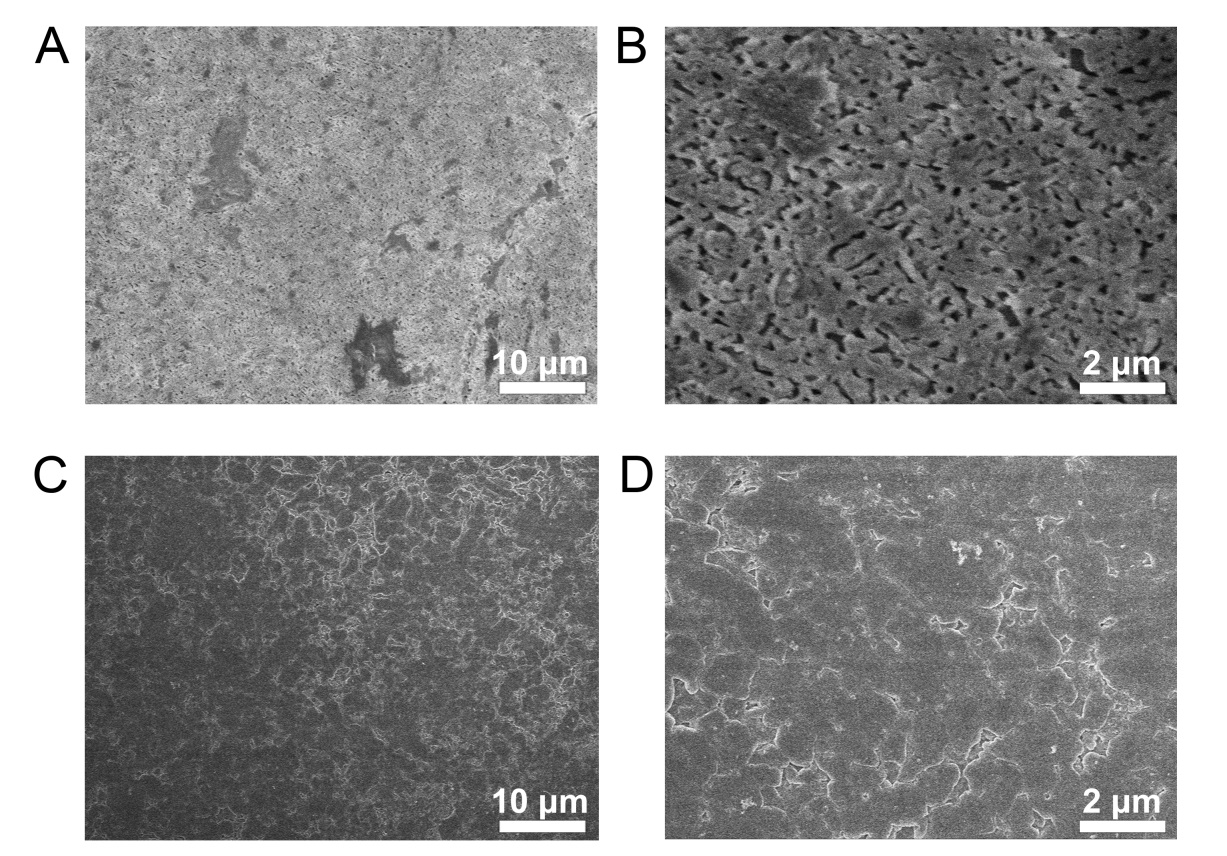


**fig. S9. SEM images of Na deposition (3 mAh) at different anodes.** (A) and (B) Al foil anode; (C) and (D) Super P/Al anode.

The morphology of Al foil and Super P/Al anode after Na deposition has been studied by the SEM. We find that 3D Na dendrites with high surface area cover the surface of Al foil. In contrast, the Na covered surface of Super P/Al anode remains smooth and no obvious 3D Na dendrite growth is discovered, which suggests a more uniform deposition process of Super P/Al anode (*5*).


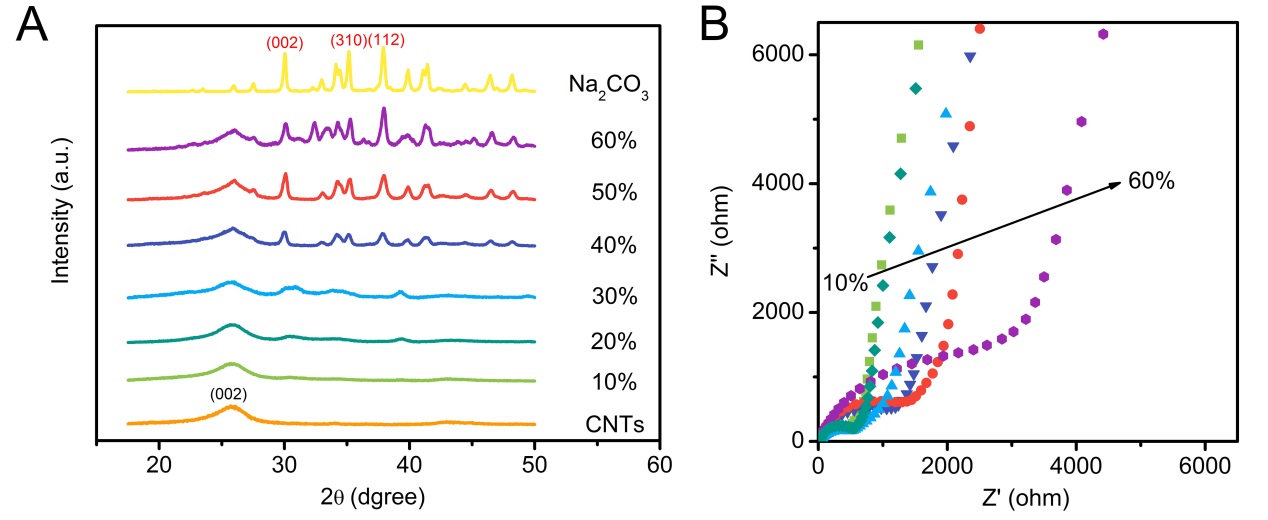


**fig. S10.** **The optimization of Na_2_CO_3_/CNTs composites.** **(A)** XRD patterns of CNTs, Na_2_CO_3_ and Na_2_CO_3_/CNTs with selected mass ratios of Na_2_CO_3_ to total Na_2_CO_3_/CNTs. **(B)** EIS of 10%, 20%, 30%, 40%, 50%, 60% (wt.) of Na_2_CO_3_ in Na_2_CO_3_/CNTs composites.


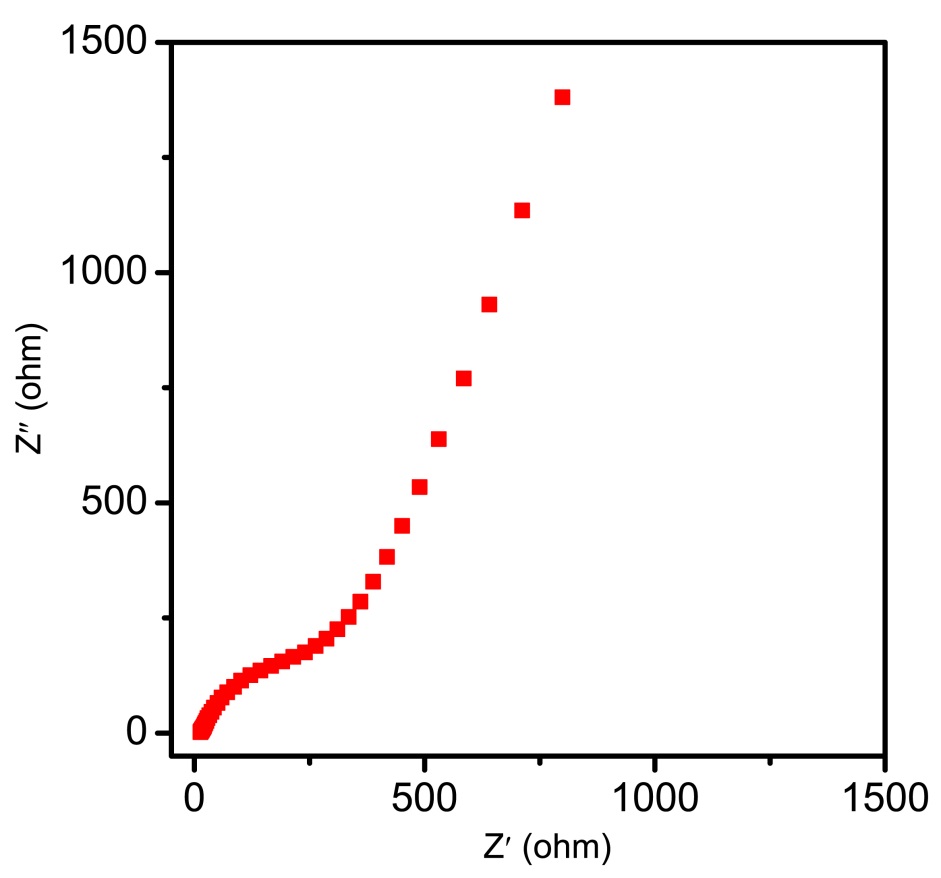


**fig. S11. EIS of mixed materials consisting of pure Na_2_CO_3_ and titanium powder (mass ratio of 1: 9).**

The density of titanium powder is high. In order to maintain the conductivity of the mixed materials, we selected the mixed materials with 10 wt% Na_2_CO_3_ for test. The addition of titanium powders can effectively reduce the resistance of the electrode.


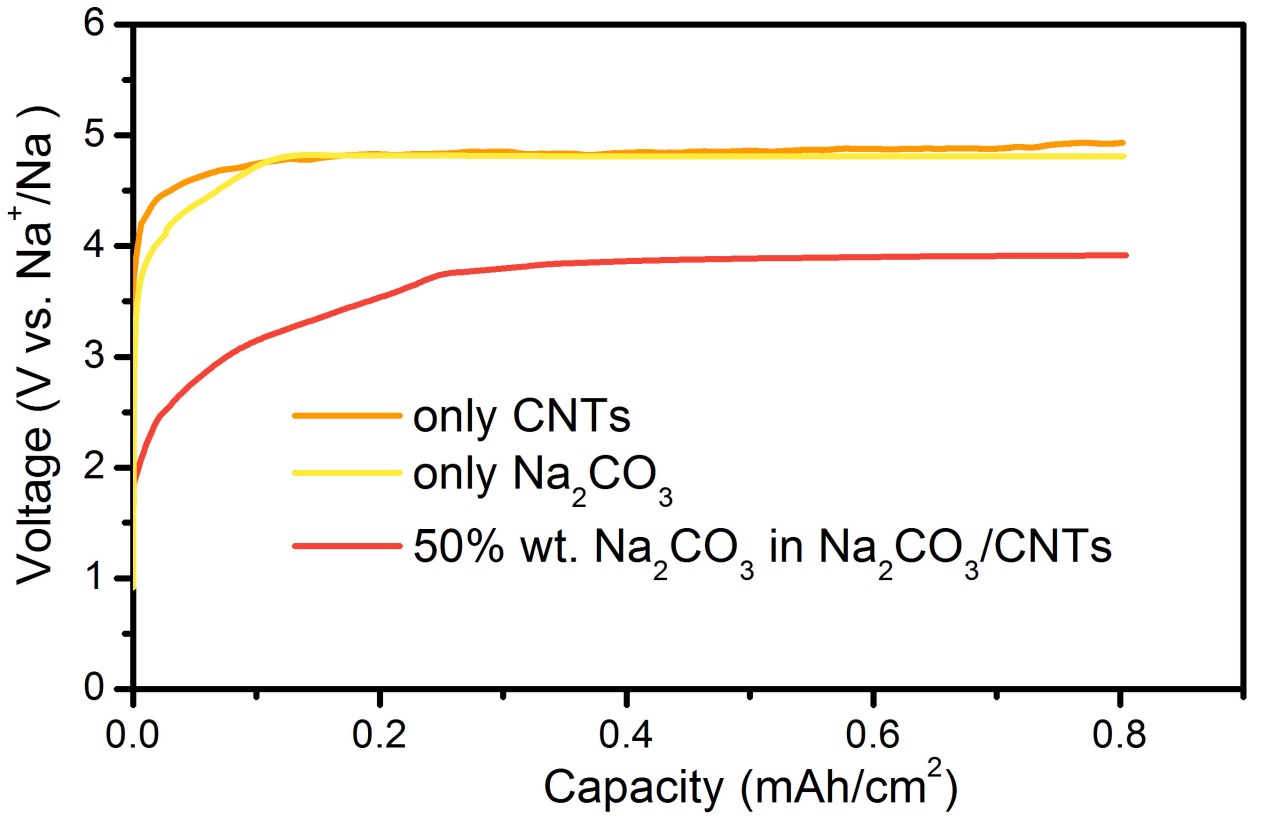


**fig. S12. The charge profiles of pure CNTs, Na_2_CO_3_/CNTs, and pure Na_2_CO_3_ with titanium powder** **at current density of 0.1 mA cm^-2^.**


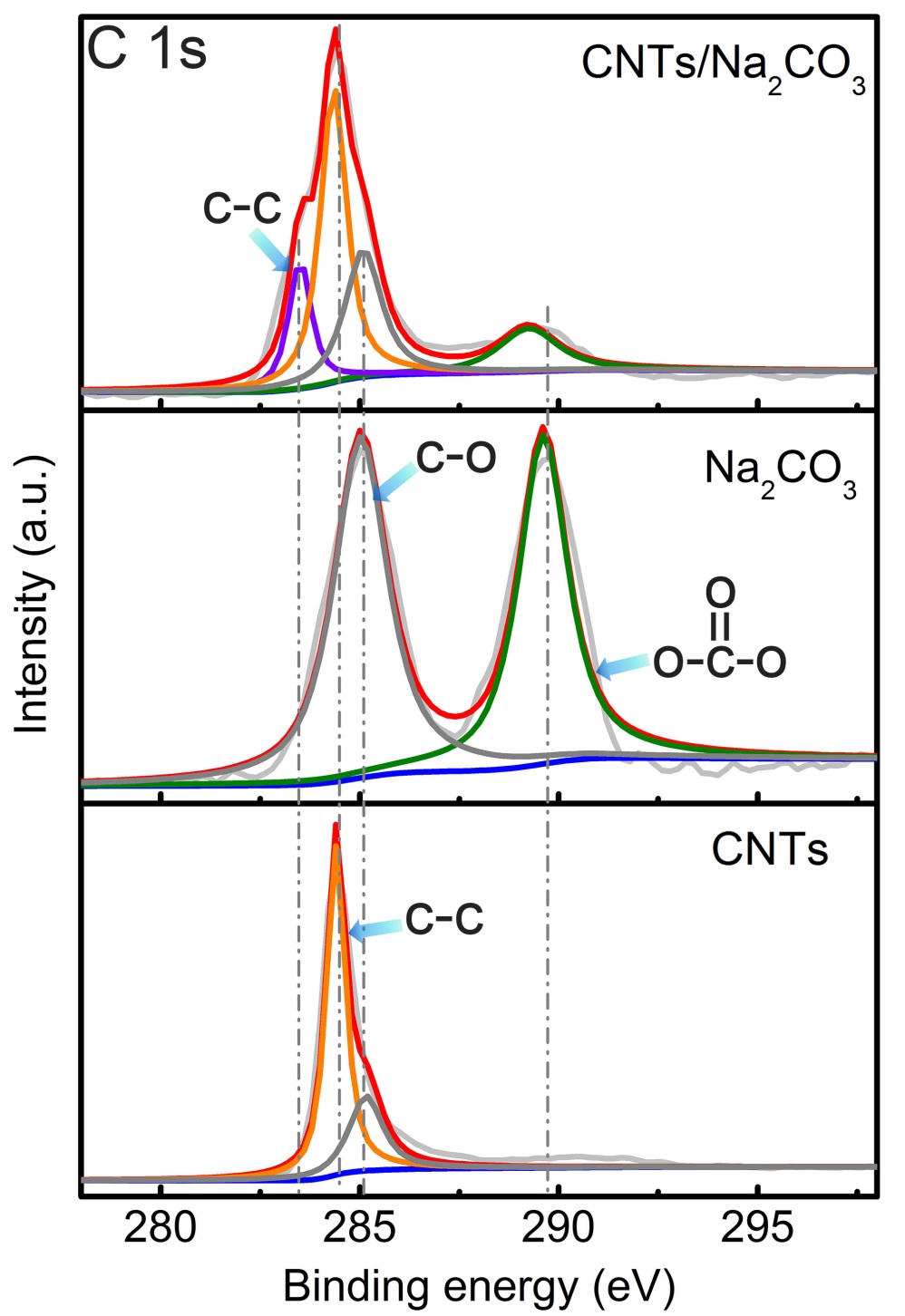


**fig. S13. The X-ray photoelectron spectroscopy (XPS) of pure CNTs, pure Na_2_CO_3_ and Na_2_CO_3_/CNTs composites cathode of 50 wt% Na_2_CO_3_ content.**


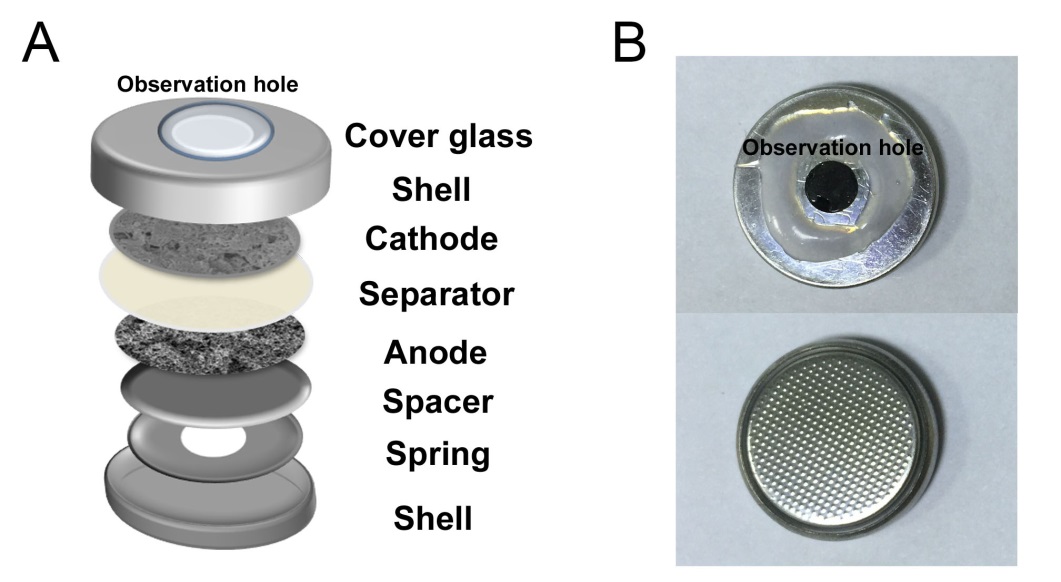


**fig. S14. The in-situ Raman battery. (A)** Schematic illustration, and **(B)** photographs of the in-situ Raman cell.


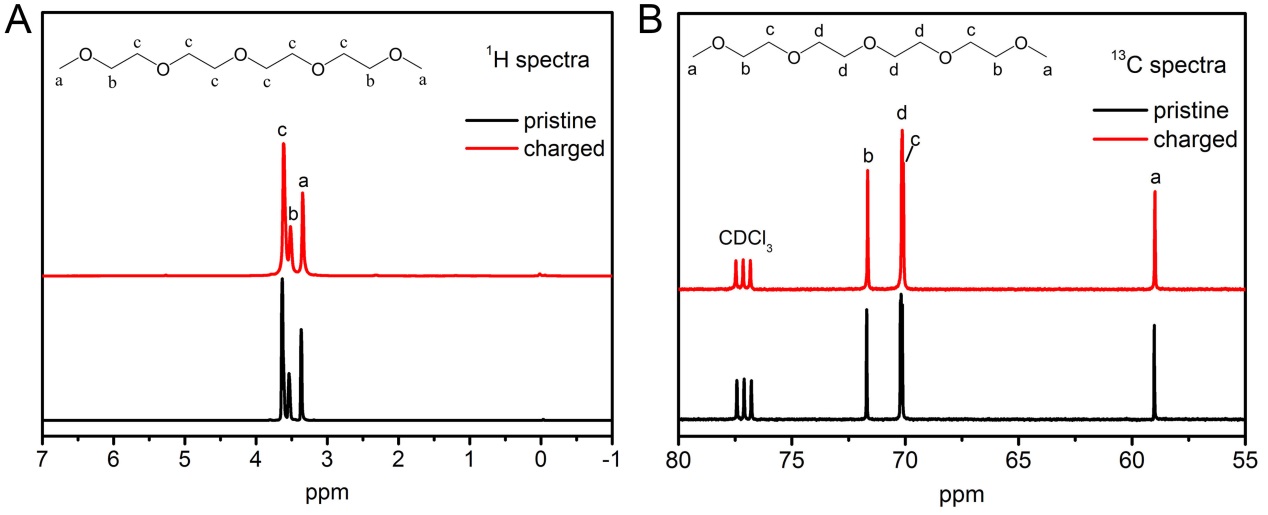


**fig. S15. ^1^H NMR and ^13^C NMR spectra of electrolyte before and after charge.**

The residual electrolyte from the cathode and separator after charge was extracted and then subjected to NMR spectroscopy with DCCl_3_ as solvent. The peaks in ^1^H and ^13^C NMR are derived from TEGDME and DCCl_3_ only. No additional peaks from decomposition products were detected.


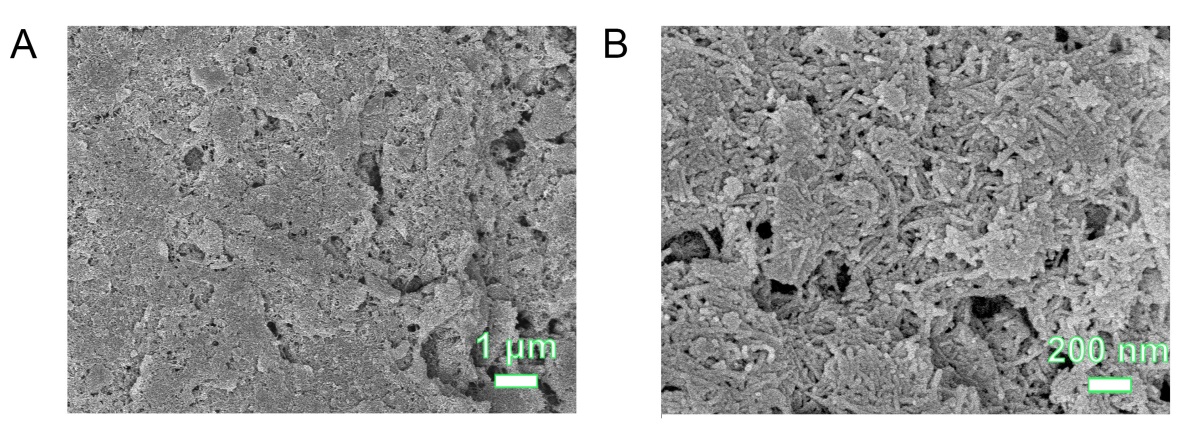


**fig. S16. SEM images of Na_2_CO_3_/CNTs cathode after charge.**


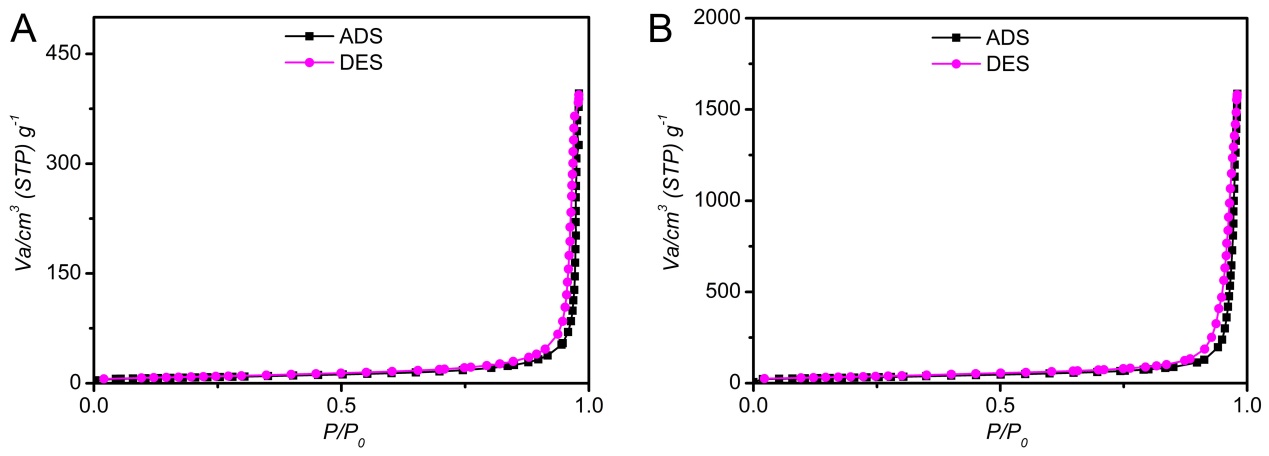


**fig. S17. The specific surface area (BET) of the cathode.** **(A)** Before and **(B)** after charge.

As the decomposition of Na_2_CO_3_ and CNTs in charging reaction, the cathode becomes porous. The specific surface area of the cathode increased from 27.498 m^2^ g^-1^ to 215.52 m^2^ g^-1^. In the pristine cathode, Na_2_CO_3_ closely combined with carbon nanotubes, resulting in a dense electrode. After charging, all the Na_2_CO_3_ is decomposed, and the original position of Na_2_CO_3_ is vacated. In addition, the carbon nanotube skeleton is well maintained. Therefore, the cathode becomes loose and porous, resulting in a significant increase in specific surface area.


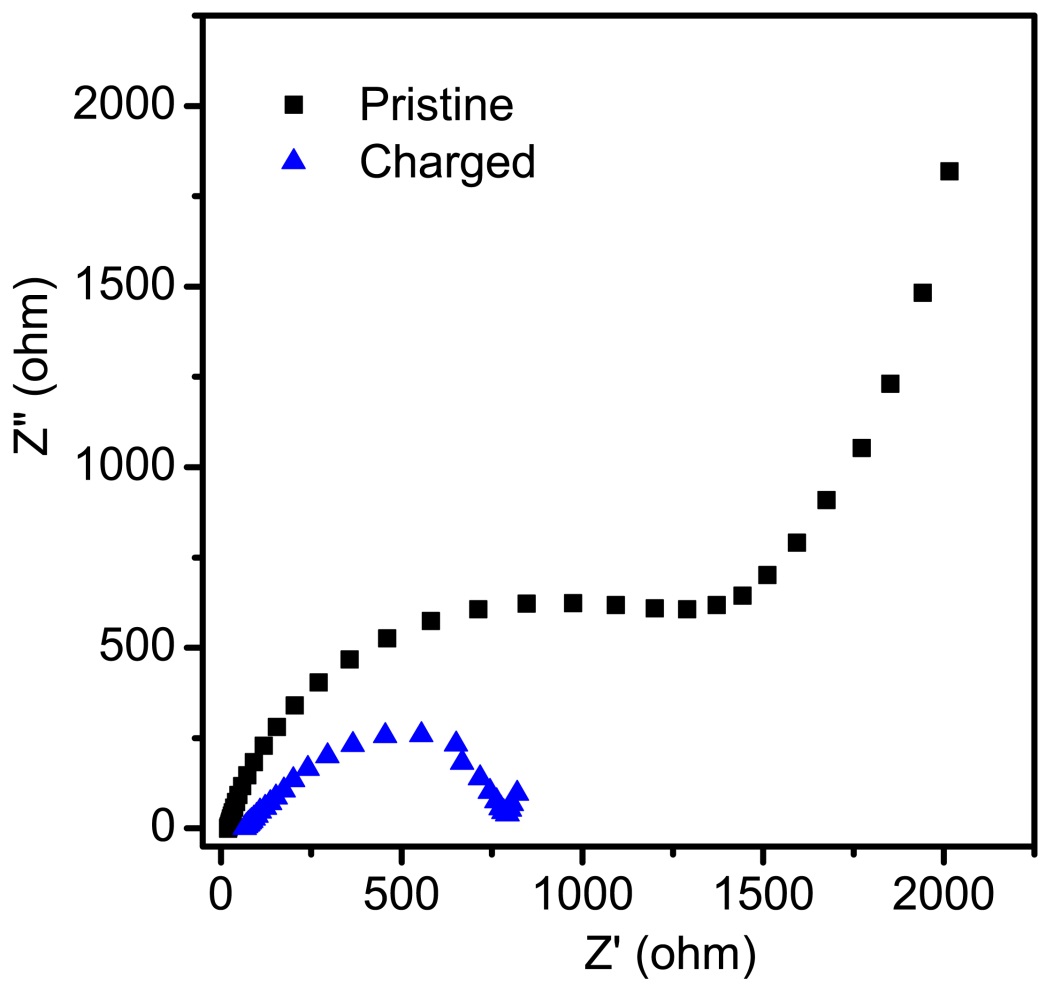


**fig. S18. EIS of the battery before and after charge.**


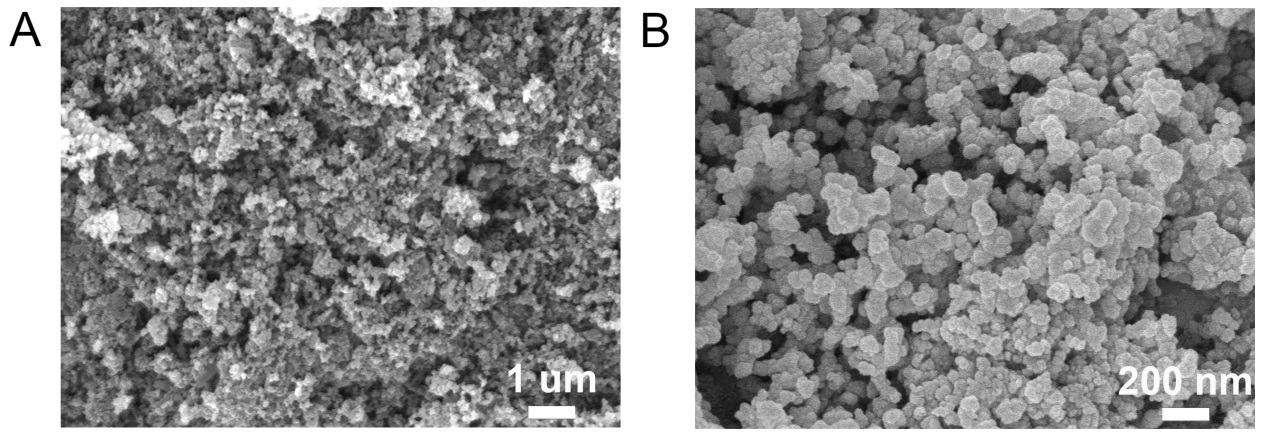


**fig. S19. SEM images of pristine Super P/Al anode.**


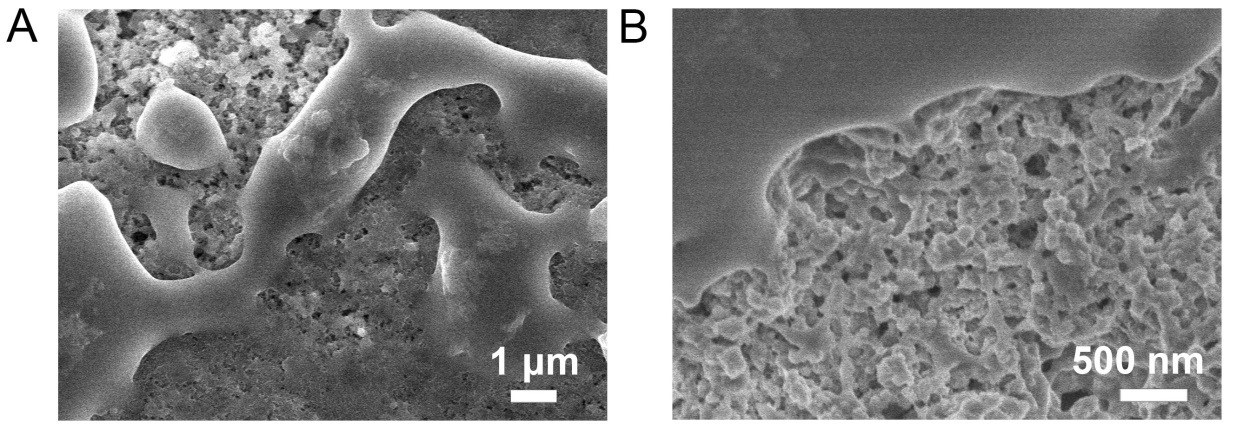


**fig. S20. SEM images of Super P/Al electrode after charging to 1 mAh.**


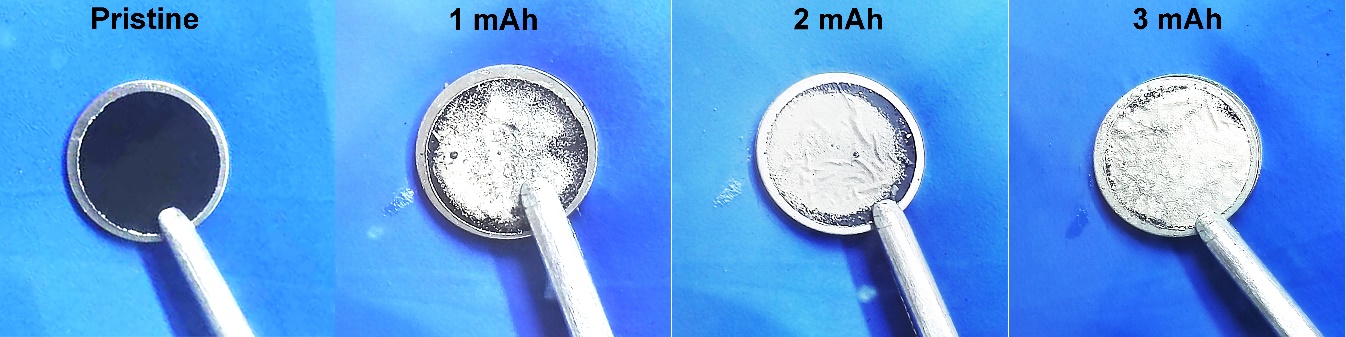


**fig. S21. The photographs of Super P/Al anode with different charging capacity (0-3 mAh).**


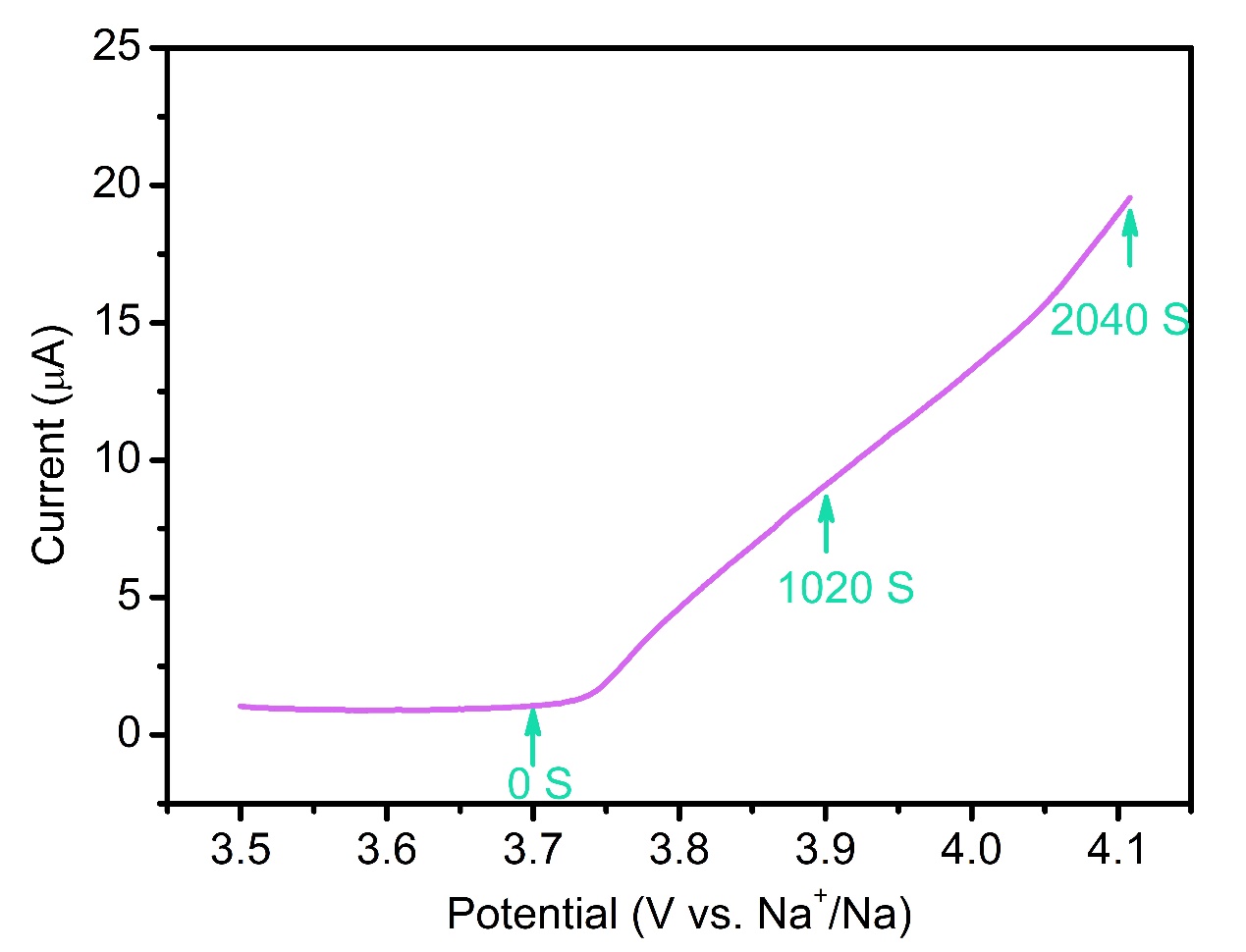
**fig. S22. A representative LSV curve of Na deposition in the in-situ** **tests with Na_2_CO_3_/CNTs as work electrode and Au as counter electrode.**


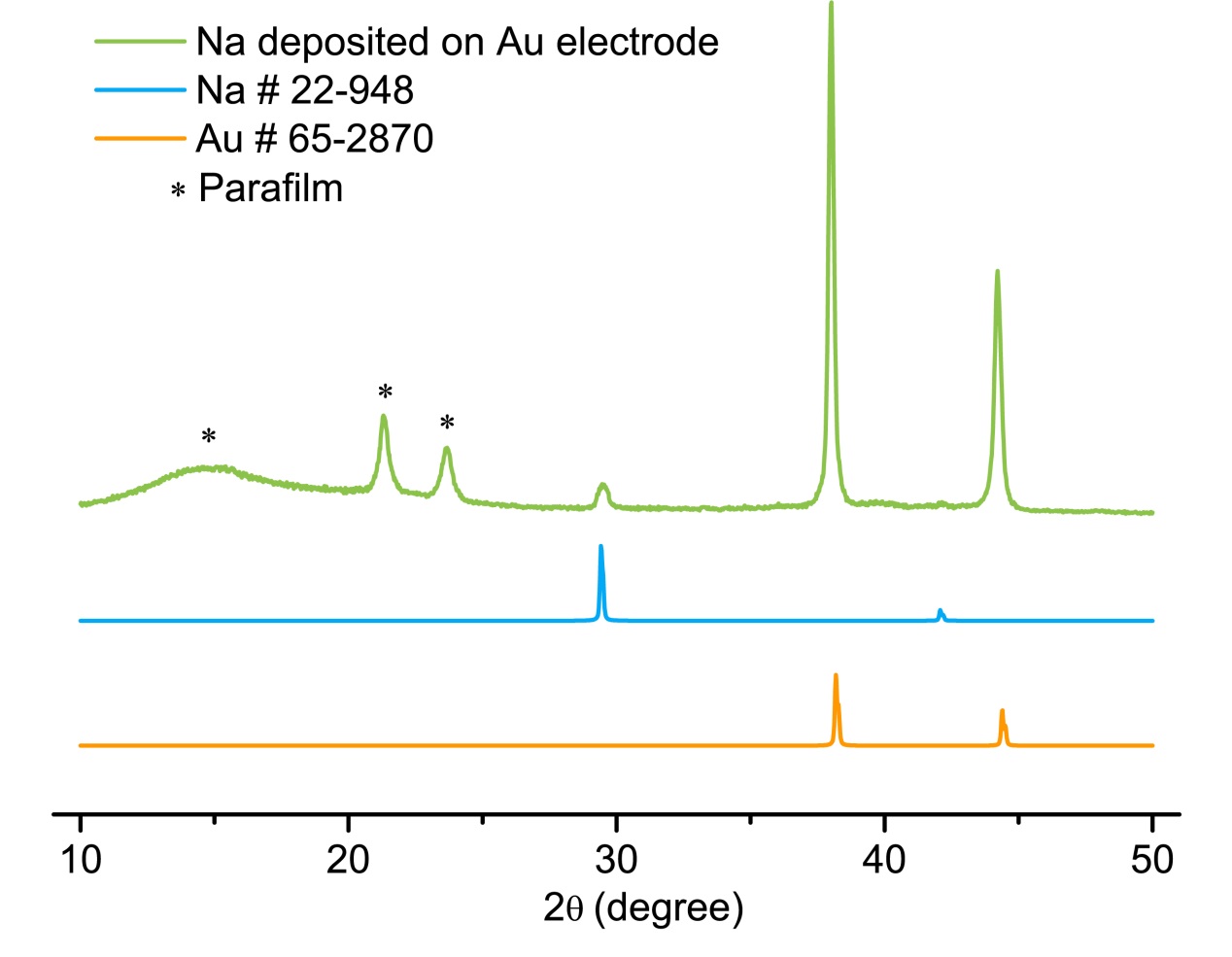


**fig. S23. XRD patterns of the deposited Na on the Au electrode.**


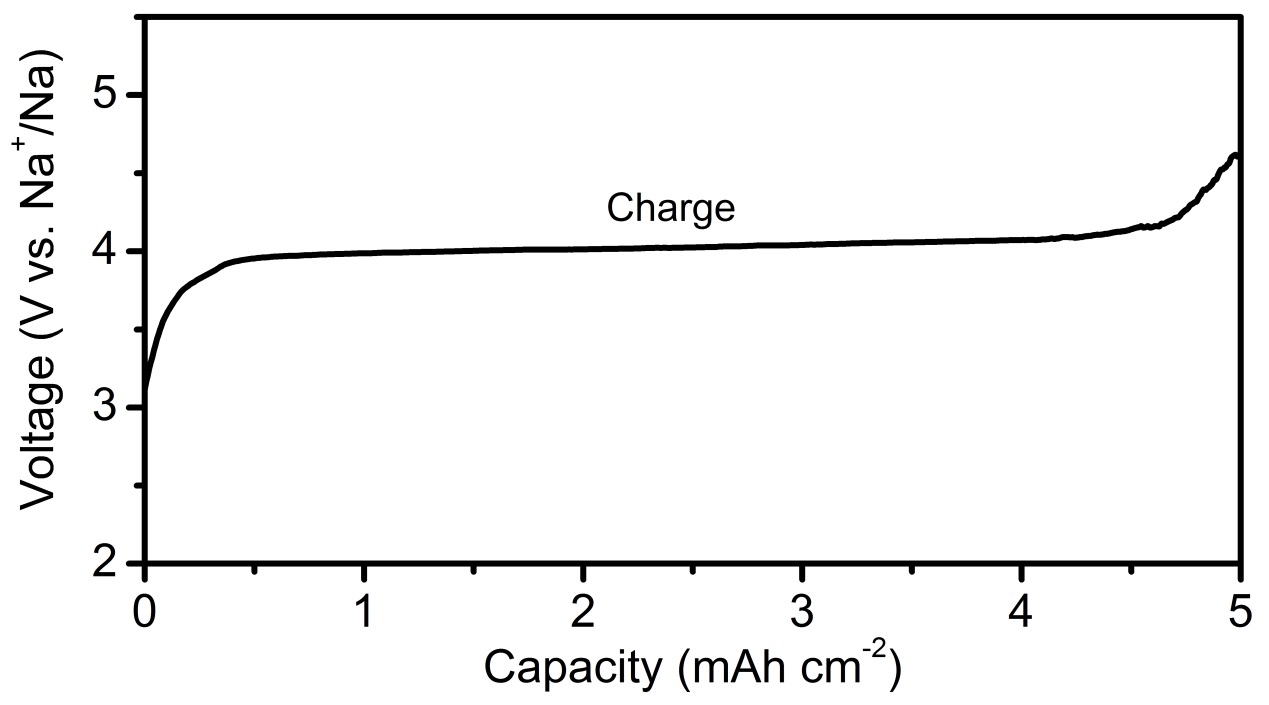


**fig. S24. The full charge profile of Na-CO_2_ batteries with 5 mAh cm^-2^ at 0.1 mA cm^-2^.**

We assemble the coin cells by using Na_2_CO_3_/CNTs cathode, Super P/Al anode, Celgard separator, and 1 M NaClO_4_/TEGDME electrolyte.


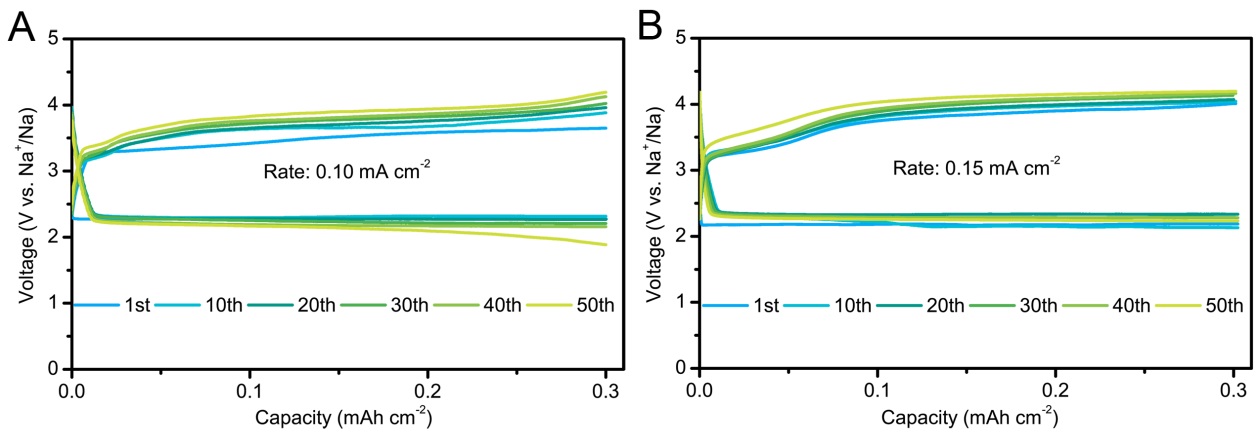


**fig. S25. The cycling stability of Na-CO_2_ batteries with a cut-off capacity of 0.3 mAh cm^-2^ at different current densities.** **(A)** 0.10 mA cm^-2^. **(B)** 0.15 mA cm^-2^.

**
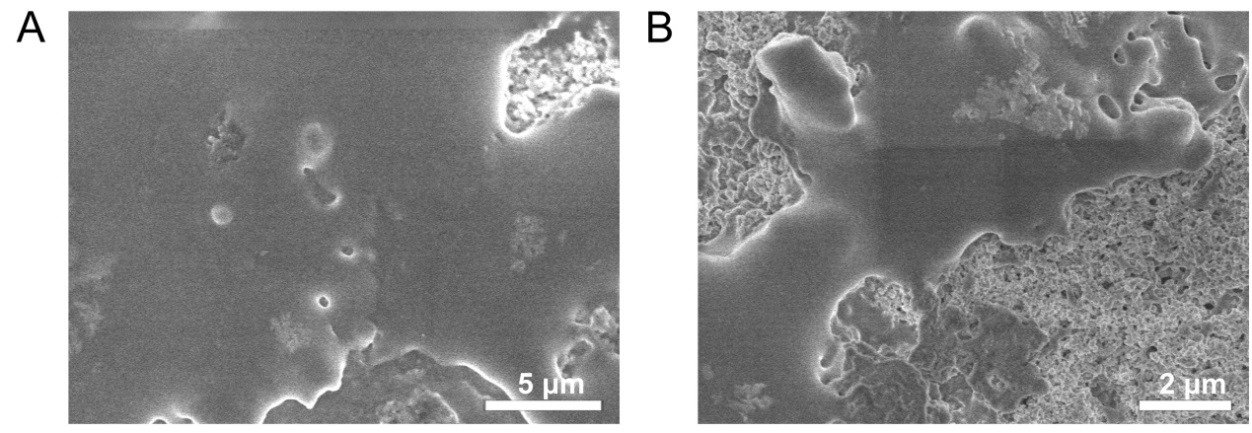
**

**fig. S26. SEM images of Na coated Super P/Al anode after 50 cycles, exhibiting a smooth surface.**

**
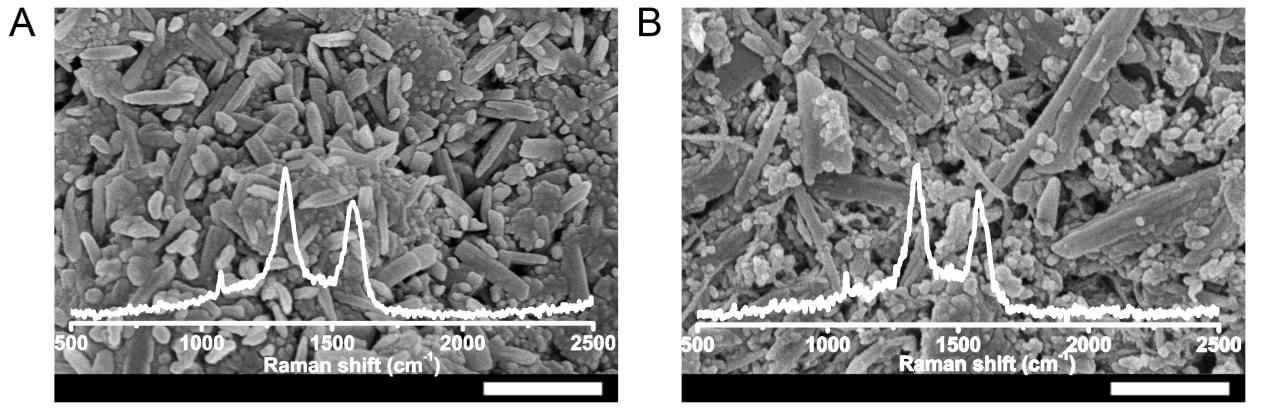
**

**fig. S27. SEM images of the discharge products after first discharge process at different rates of (A) 0.10 mA cm^-2^ and (B) 0.15 mA cm^-2^.** The insets in (A) and (B) are the corresponding Raman spectrum. Scale bar: 500 nm.


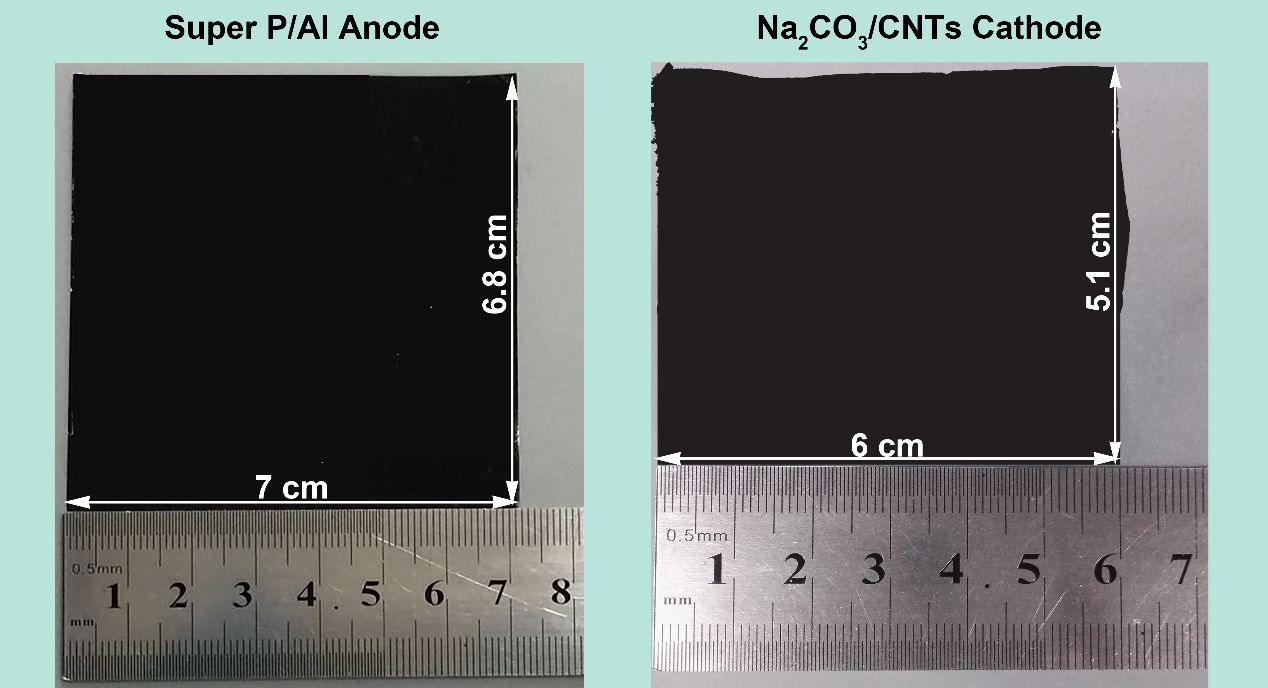


**fig. S28. Photographs of Super P/Al anode and Na_2_CO_3_/CNTs cathode.**

One large Na_2_CO_3_/CNTs cathode (6 × 5.1 cm^2^, 1.6 g), a Super P/Al anode (7 × 6.8 cm^2^, 0.25g), and a Celgard separator (8 × 7 cm^2^) were fabricated in a plastic mold.


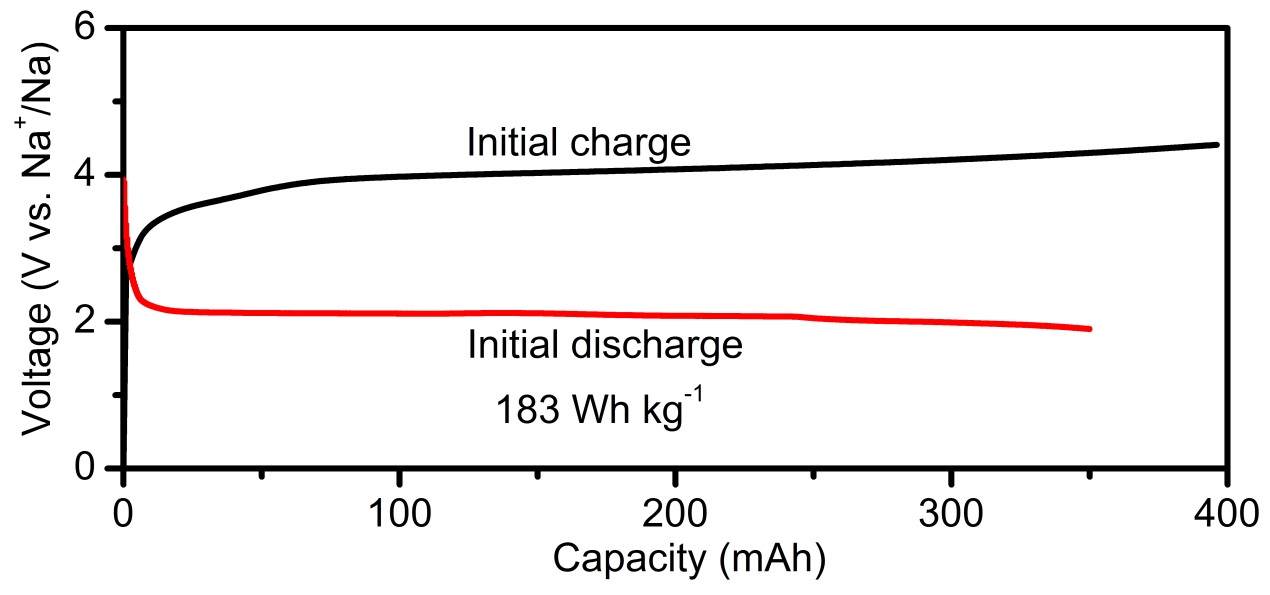


**fig. S29. Pouch-type battery performance.** Initial full charge/discharge profiles. Current:10 mA.

**movie S1. Sodium deposition process**

**movie S2. Process that bulb is lit up**

**References**

37. S. A. Freunberger, Y. H. Chen, N. E. Drewett, L. J. Hardwick, F. Barde, P. G. Bruce, The lithium-oxygen battery with ether-based electrolytes. *Angew. Chem. Int. Ed.* **50**, 8609-8613 (2011).

38. A. C. Luntz, B. D. McCloskey, Nonaqueous Li-air batteries: a status report. *Chem. Rev.* **114**, 11721-11750 (2014).

39. N. Song, H. Liu, J. Fang, Fabrication and mechanical properties of multi-walled carbon nanotube reinforced reaction bonded silicon carbide composites. *Ceram. Int.* **42**, 351-356 (2016).

40. K. Zhang, G. H. Lee, M. Park, W. Li, Y. M. Kang, Recent developments of the lithium metal anode for rechargeable non-aqueous batteries. *Adv. Energy Mater.* **6**, 1600811 (2016).
